# Cohesin Rings Devoid of Scc3 and Pds5 Maintain Their Stable Association with the DNA

**DOI:** 10.1371/journal.pgen.1002856

**Published:** 2012-08-09

**Authors:** Irina Kulemzina, Martin R. Schumacher, Vikash Verma, Jochen Reiter, Janina Metzler, Antonio Virgilio Failla, Christa Lanz, Vipin T. Sreedharan, Gunnar Rätsch, Dmitri Ivanov

**Affiliations:** 1 Friedrich Miescher Laboratory of the Max Planck Society, Tübingen, Germany; 2 Max Planck Institute for Developmental Biology, Tübingen, Germany; Duke University, United States of America

## Abstract

Cohesin is a protein complex that forms a ring around sister chromatids thus holding them together. The ring is composed of three proteins: Smc1, Smc3 and Scc1. The roles of three additional proteins that associate with the ring, Scc3, Pds5 and Wpl1, are not well understood. It has been proposed that these three factors form a complex that stabilizes the ring and prevents it from opening. This activity promotes sister chromatid cohesion but at the same time poses an obstacle for the initial entrapment of sister DNAs. This hindrance to cohesion establishment is overcome during DNA replication via acetylation of the Smc3 subunit by the Eco1 acetyltransferase. However, the full mechanistic consequences of Smc3 acetylation remain unknown. In the current work, we test the requirement of Scc3 and Pds5 for the stable association of cohesin with DNA. We investigated the consequences of Scc3 and Pds5 depletion *in vivo* using degron tagging in budding yeast. The previously described DHFR–based N-terminal degron as well as a novel Eco1-derived C-terminal degron were employed in our study. Scc3 and Pds5 associate with cohesin complexes independently of each other and require the Scc1 “core” subunit for their association with chromosomes. Contrary to previous data for Scc1 downregulation, depletion of either Scc3 or Pds5 had a strong effect on sister chromatid cohesion but not on cohesin binding to DNA. Quantity, stability and genome-wide distribution of cohesin complexes remained mostly unchanged after the depletion of Scc3 and Pds5. Our findings are inconsistent with a previously proposed model that Scc3 and Pds5 are cohesin maintenance factors required for cohesin ring stability or for maintaining its association with DNA. We propose that Scc3 and Pds5 specifically function during cohesion establishment in S phase.

## Author Summary

When a cell divides, each daughter cell receives one, and only one, of each sister DNA molecule from the mother. These identical DNA molecules, called chromatids, result from the replication of a single DNA molecule and are held together by a ring-shaped protein complex termed cohesin. As a cell’s genetic information is divided into several distinct chromosomes, this arrangement, termed sister chromatid cohesion, makes it possible to distinguish sister and non-sister chromatids and is a prerequisite for the faithful division of genetic information. Cohesin rings, consisting of three subunits, trap two sister DNA molecules inside them. Additional proteins are required to load the rings onto DNA and to ensure that they capture both sister DNA molecules. We have investigated the roles of Scc3 and Pds5, two proteins that associate with cohesin rings, and were previously proposed to keep them stably locked once loaded onto DNA. Surprisingly, when we depleted Scc3 and Pds5 from yeast, the rings remained stably associated with the DNA; however, cohesion between the sisters was severely compromised. We conclude that Scc3 and Pds5 function to capture the two sister DNA molecules together inside the cohesin ring.

## Introduction

Cohesin is a ring-shaped protein complex whose major function is to hold sister chromatids together from the onset of DNA replication until their separation to daughter cells in anaphase of mitosis (for review see [1]). The cohesin ring is composed of Smc1, Smc3 and Scc1. At least three additional proteins, Scc3, Pds5, and Wpl1, associate with the ring. Smc1 and Smc3 both contain a 50 nm long intramolecular anti-parallel coiled coil flanked by a central hinge domain on one side and, on the other, by an ATPase head domain formed from the N and C-terminal regions of the protein. The hinge domain of Smc1 associates with the hinge domain of Smc3. Connecting the two head domains is Scc1, thus completing the ring.

Cohesin was recently demonstrated to function by capturing two sister DNAs inside a single ring [2] although alternative models have also been proposed [3]. However, the ring is also capable of embracing a single sister, which does not lead to the establishment of sister chromatid cohesion [4]. Stable capture of both sisters is ensured via the action of an acetyltransferase, Eco1 [5]–[7]. Eco1 acetylates two adjacent lysine residues in the ATPase head domain of Smc3, which in budding yeast correspond to lysines 112 and 113 [8]–[10]. Mutation of both lysines to non-acetylatable arginines is lethal while their mutation to acetylation-mimicking asparagines or glutamines makes Eco1 dispensable for cohesion establishment. The relevant target of Eco1 acetylation in S phase differs from acetylation in response to double-stranded DNA breaks when two lysine residues of Scc1, K84 and K210, are proposed to be critical [11]. Acetylation of cohesin is initiated during S phase after it is loaded onto DNA and persists through G2 until cell division. Acetylated cohesin can only inefficiently establish cohesion, necessitating either de novo synthesis of non-acetylated Smc3 or deacetylation of the Smc3 that was released from DNA in the previous mitotic cycle. A deacetylase, Hos1 was recently discovered to be critical for Smc3 deacetylation [12]–[14].

The mechanistic role of cohesin acetylation remains unclear. It is reported to counteract the function of Wpl1, also known in budding yeast as Rad61 [9], [15]. While Wpl1 function in yeast remains to be established, the vertebrate Wpl1 orthologue is required for the removal of cohesin from DNA in prophase of mitosis [16], [17]. Wpl1 forms a complex with Scc3 and Pds5 *in vitro* and mutations in *WPL1*, *SCC3* and *PDS5* genes were found to suppress lethality caused by *eco1* deletion [18]. *SCC3* [6] and *PDS5* [19], [20] were discovered in yeast as genes that when mutated result in cohesion defects. Both proteins are conserved in evolution from yeast to humans [21]–[23]. In budding yeast both *SCC3* and *PDS5* are essential. However, in fission yeast *PDS5* can be deleted [24], reflecting different requirements for Pds5 function in different organisms. In budding yeast, Pds5 is comprised of 26 HEAT repeats and a highly charged C-terminal domain [19]. Scc3 was also predicted to contain HEAT repeats [25], although they appear too divergent to be predicted with statistical confidence. The phosphorylation of Scc3 in mammalian mitosis leads to the Wpl1-dependent removal of cohesin from chromosomes [26]. Therefore it appears that all three proteins are, at least in some organisms, involved in destabilizing the association of cohesin with DNA. On the other hand, Scc3 and Pds5 have been proposed to be cohesin maintenance factors because temperature-sensitive mutations in the respective genes result in reduced association of cohesin with DNA [6], [19]. Importantly, Eco1 mediated cohesin acetylation is implicated in the stabilization of cohesin binding to DNA during S phase [27] and at the same time it is thought to counteract the function of Scc3, Pds5 and Wpl1 [18].

Early studies of Scc3 and Pds5 function in vivo relied on conditional mutations that inactivate the proteins at the restrictive temperature of 37°C. These mutants frequently contain multiple amino acid substitutions and the mechanism by which they affect protein function is therefore difficult to elucidate. We decided to deplete Scc3 and Pds5 from the cell by fusing the genes to degron sequences. A “conventional” DHFR-based temperature-induced degron fused to Scc3 caused the degradation of Scc1, a “core” subunit of cohesin complex precluding the specific analysis of the role of Scc3. However, using a novel and efficient degron derived from the Eco1 protein we were able to specifically deplete Scc3 and Pds5 in the cell while leaving the “core” cohesin subunits intact. Our results demonstrate that in the absence of Scc3 and Pds5, cohesin rings remain stably associated with chromosomes. Moreover, their distribution throughout yeast genome remains unaffected. However, the destabilization of Scc3 and Pds5 significantly weakens sister chromatid cohesion and causes chromosomal mis-segregation, suggesting that the essential function of these proteins is in cohesion establishment rather than the maintenance of cohesin on DNA.

## Results

### Interaction of Scc3, Pds5 and Wpl1 with the cohesin ring subunits in vitro

The arrangement of Scc3 and Pds5 proteins within cohesin complex is poorly understood. Recombinant yeast and human Scc3 proteins were reported to interact respectively with the C-terminal part of Scc1 [28] or central region of Scc1 which is poorly conserved between yeast and humans [29]. Recently, it was reported that a deletion of nine amino acids (319–327) at the end of the central region of Scc1 in budding yeast disrupts its association with Scc3 in vivo [30]. It has also been proposed that Scc3 facilitates the interaction between Pds5/Wpl1 and the cohesin ring [29].

**Figure 1:**
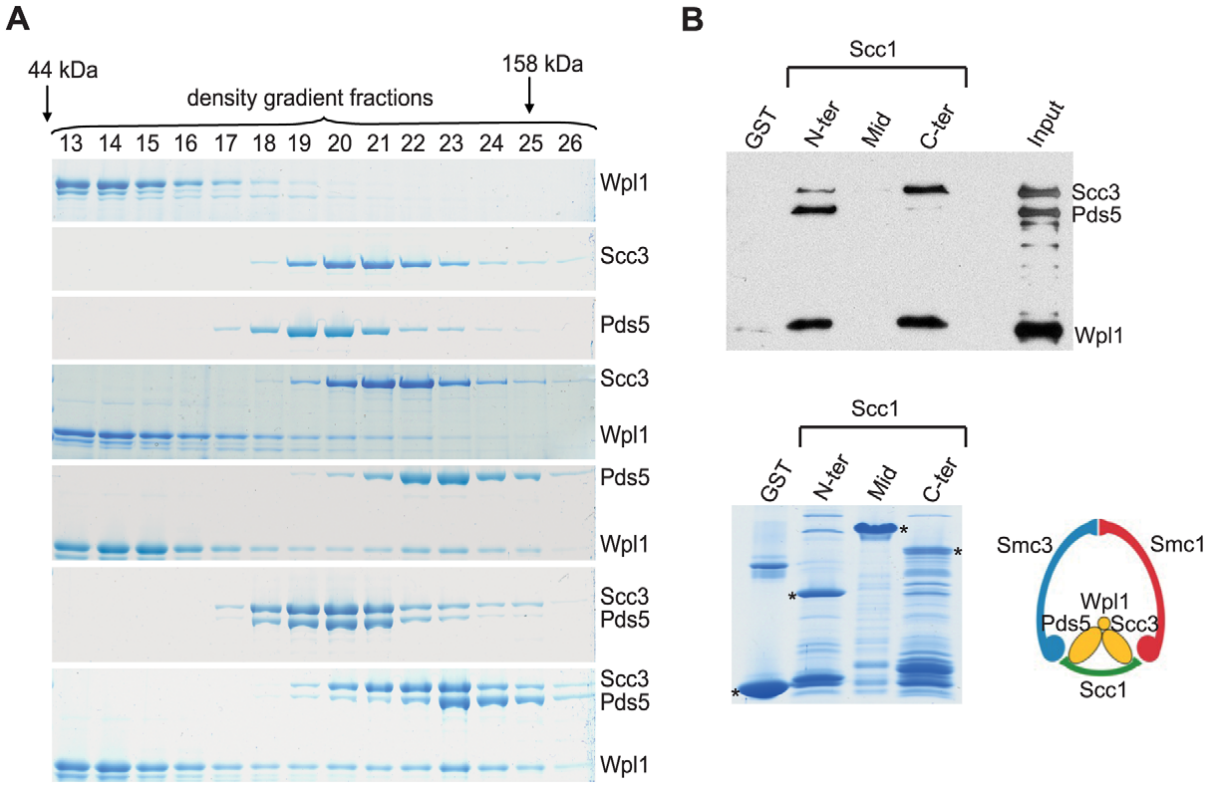
Scc3, Pds5, and Wpl1 form a complex and associate with Scc1 “core” subunit of cohesin. (A) Purified recombinant Scc3, Pds5 and Wpl1 proteins were mixed as indicated and separated by ultracentrifugation on a 10–30% glycerol gradient. A total of 44 gradient fractions were collected and analyzed on a Coomassie-stained 6% SDS-PAGE. Only fractions containing the proteins are shown. Positions of molecular size markers are indicated. (B) Recombinant His6-tagged Scc3, Pds5, and Wpl1 were mixed together and incubated with glutathione-agarose beads charged with GST or GST-fused to the N-terminal (aa 1–168), middle (aa 169–337) or C-terminal (aa 338–566) regions of Scc1. Beads eluates were analyzed by Western blotting with Penta-His antibody (QIAGEN) (upper panel). Coomassie-stained GST-beads are shown in the lower panel.

We investigated interactions of Scc3, Pds5 and Wpl1 with cohesin ring subunits using glycerol gradient centrifugation of recombinant yeast proteins purified from *E. coli*. All proteins were purified using gel filtration chromatography and were confirmed not to be aggregated. In agreement with a previous study [18], we were able to detect a strong interaction between Wpl1 and Pds5. Both proteins migrate in the same fractions, which are significantly faster than Pds5 alone, the larger of the two subunits (Figure 1A). Binding between Scc3 and Wpl1 appeared to be weaker. Wpl1 was detected in the later fractions containing Scc3 but there was little change in Scc3 migration on the gradient possibly due to low stability of the complex. Addition of Scc3 to the Pds5-Wpl1 complex resulted in a further shift towards the bottom of the gradient consistent with the formation of a trimeric complex, which has been previously proposed [18]. However, this shift was very small considering an expected 1.7 fold increase in the estimated molecular weight of the trimeric complex (354 kDa) compared to the Pds5/Wpl1 dimer (221 kDa). It is possible that the Scc3/Pds5/Wpl1 complex assumes a very extended conformation that results in unexpectedly slow migration through glycerol gradients. Alternatively, Scc3 might only weakly associate with the complex making the interaction too unstable to survive the long centrifugation.

We next explored interactions between the subunits of the cohesin complex and Scc3, Pds5, and Wpl1 in vitro using gradient centrifugation. We could not detect any interaction of these cohesin-associated proteins with the Smc1 and Smc3 head and hinge domains or with the Smc3 coiled coil (data not shown). Because recombinant Scc1 has poor solubility and could not be used in gradient centrifugation experiments, we expressed its N-terminal, middle and C-terminal regions as GST-fusions and employed a GST pull-down assay to test their interaction with the Scc3, Pds5 and Wpl1. The Scc3/Wpl1complex was found to interact with the C-terminal part of Scc1 (Figure 1B), consistent with an earlier study [28]. Interestingly, the Pds5/Wpl1 complex bound to the N-terminal region of Scc1 (Figure 1B), which has previously been demonstrated to interact with the Smc3 head [28]. Remarkably, although Scc3, Pds5, and Wpl1 were mixed together in these experiments, they interacted with Scc1 as separate Scc3/Wpl1 and Pds5/Wpl1 heterodimers rather than as a single trimeric complex. Accordingly, only a small amount of Scc3 was detected in the pull-down with N-terminal region of Scc1 and very little Pds5 was found to interact with Scc1 C-terminus. These observations further highlight the poor stability of the Scc3/Pds5/Wpl1 complex, even at the low salt concentrations that were used in our experiments.

The interaction sites of Scc1 that we observed for Scc3 and Pds5 suggest a possible role for these proteins in stabilizing the cohesin ring. Pds5 bound close to the interface between the Scc1 N-terminal domain and the Smc3 head while Scc3 binding was adjacent to the interface between the Scc1 C-terminal domain and the Smc1 head. Thus, Pds5 and Scc3 can potentially re-enforce the interaction of Scc1 with Smc's and/or affect the interaction between the Smc heads, which is required for ATP hydrolysis and opening of the hinge during cohesin loading on DNA [31].

### Pds5 depletion has no effect on cohesin association with chromosomes

**Figure 2:**
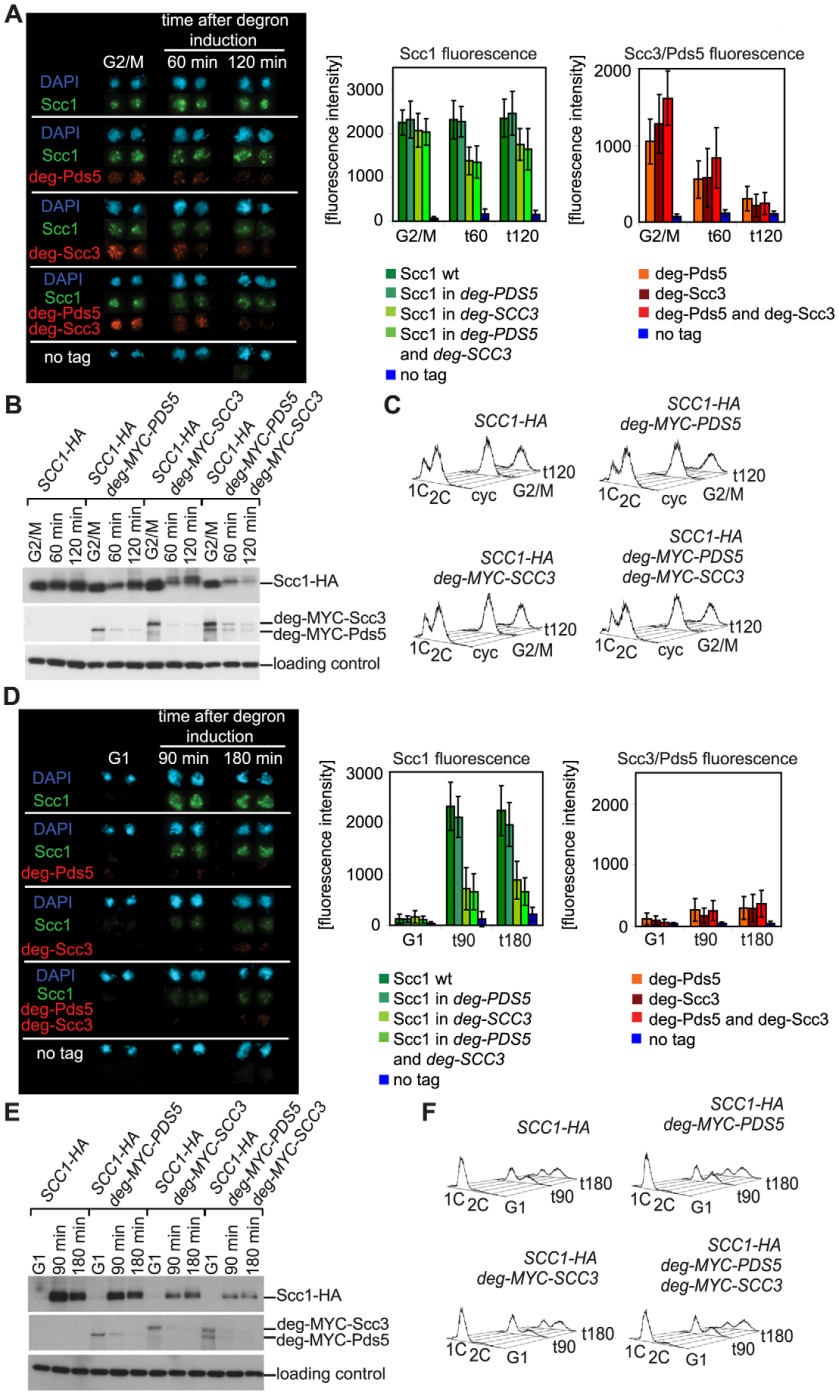
Depletion of Scc3 and Pds5 with a “conventional” temperature-sensitive degron. (A–C) Strains 2395 (*SCC1-HA6*), 2452 (*SCC1-HA6*, degron-*MYC18-PDS5*), 2455 (*SCC1-HA6*, degron-*MYC18*-*SCC3*) and 2456 (*SCC1-HA6*, degron-*MYC18- PDS5, degron-MYC18-SCC3*) were arrested with nocodazole in YEP raffinose at 30°C for 2 hours, resuspended in YEP galactose containing nocodazole and incubated for 45 minutes at 30°C to induce the expression of Ubr1. Cells were shifted to 37°C in YEP galactose containing nocodazole and doxycycline to deplete Pds5 and/or Scc3. (A) Chromosomal spreads were prepared at the indicated time points and stained with DAPI for DNA, anti-HA (mouse, 16B12) and anti-MYC (rabbit, 71D10) antibodies. The secondary antibodies were Alexa Fluor 488 anti-mouse and Alexa Fluor 568 anti-rabbit. Protein fluorescence was quantified using Metamorph software. At every time point fluorescence of 50 nuclei was determined. Error bars represent standard deviation. (B) Western blot of TCA protein extracts probed with anti-HA (16B12), anti-MYC (71D10) and anti-Cdc28 (sc-28550, Santa Cruz). (C) FACS analysis of cellular DNA content. (D–F) Strains were staged in G1 with *α*-factor in YEP raffinose at 30°C, resuspended in YEP galactose containing *α*-factor and incubated for 45 minutes at 30°C to induce the expression of Ubr1. Cells were then shifted to 37°C in YEP galactose containing doxycycline and *α*-factor, incubated for 90 minutes to deplete Pds5 and/or Scc3 and subsequently released in YEP galactose containing nocodazole and doxycycline at 37°C. Chromosomal spreads (D), Western blot (E), and FACS analysis of cellular DNA content (F) are shown.

In order to examine whether Scc3 and Pds5 stabilize cohesin rings on DNA in vivo we wanted to deplete them from yeast cells. Since both proteins are essential in budding yeast, we constructed *SCC3* and *PDS5* gene fusions to an N-terminal “degron” sequence. In these experiments we utilized a “conventional” DHFR-based degron in a strain that overexpresses an ubiquitine ligase Ubr1 from the *GAL1* promoter [32], [33]. Upon shifting to 37°C the degron unfolds and is recognized by Ubr1, which leads to ubiquitinylation and degradation of the target protein. The target gene is placed under the control of the *CUP1* promoter which is shut down in the absence of CuSO_4_ in the medium. When applied to Scc3 and Pds5, this “conventional” degron approach resulted in a very moderate decrease in protein abundance and strains were able to grow at 37°C on galactose-containing medium without CuSO_4_ (data not shown). Therefore we utilized an alternative approach to silence *SCC3* and *PDS5* transcription described in [34]. Tet operator sequences were introduced in the promoter. In the absence of doxycycline, transactivator (tTA) activates transcription of the gene while in the presence of doxycycline a Tet repressor (tetR'-SSN6) replaces transactivator and silences transcription. This approach resulted in an efficient depletion of Scc3 and Pds5 proteins in the cell, as judged by Western blot analysis (Figure S1) and chromosomal spreads (Figure 2). Strains were unable to grow at 37°C on galactose-containing medium with doxycycline. However, protein levels of Scc1, a “core” subunit of cohesin complex, were also reduced when the degron was induced. The reduction of Scc1 abundance was only modest in the case of Pds5 but very significant when Scc3 was destabilized (Figure 2B and E). This result implied that the Scc3-degron targets Scc1 for degradation and precluded further analysis of the role of Scc3 in vivo using a “conventional” degron approach. Surprisingly, the destruction of Pds5 had little or no effect on the amount of Scc1 detected in chromosomal spreads regardless of whether the degron was induced in cells arrested in G2 with nocodazole (Figure 2A) or already in G1 prior to cohesin loading on DNA (Figure 2D). Destruction of Pds5 in a single cycle experiment had little effect on sister chromatid cohesion regardless of when the degron was induced (Figure S2).

### The Eco1 protein contains degron sequences that can be used to specifically deplete Scc3 and Pds5 from the cell

**Figure 3:**
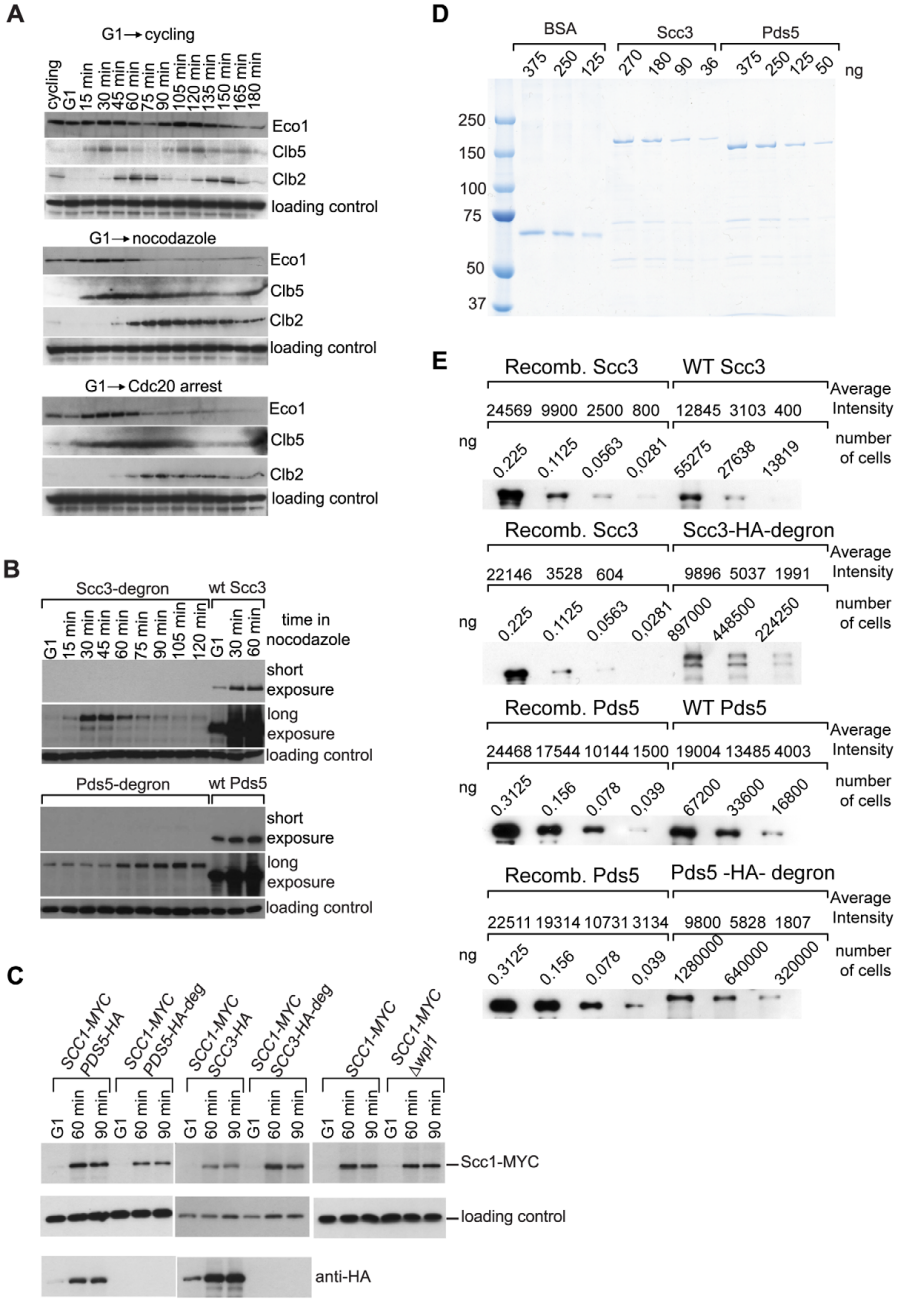
Eco1 contains a degron. (A) 1222 strain with *CDC20* expressed from a methionine-repressible promoter (*ECO1-TAP*) was staged in G1 with *α*-factor and then released into three different media. Release into the medium without methionine allows cell cycling (top) while release into full medium with methionine or nocodazole (middle and bottom) results in progression through replication and eventual G2 arrest. Expression of S phase cyclin Clb5 is induced during DNA replication while cyclin Clb2 accumulates in G2 and is destroyed in mitosis. TCA protein extracts were prepared and analyzed by Western blot. Eco1 was detected with peroxidase-anti-peroxidase, Clb5 with sc-6704, Clb2 with sc-9071, loading control with anti-Cdc28 (sc-28550, Santa Cruz). (B) Depletion of Scc3 and Pds5 with an Eco1-derived degron. G1-staged strains 12544 (*SCC3-HA6*), 1323 (*SCC3-HA6*-degron), 1677 (*PDS5-HA6*) and 1675 (*SCC3-HA6*-degron) were released into full media with nocodazole. Western blot was probed with anti-HA antibody. (C) Scc1 protein level is unchanged in the strains with Eco1-derived degron. Strains 1815 (*SCC1-Myc18*, *PDS5-HA6*), 1818 (*SCC1-Myc18*, *PDS5-HA6*-degron), 1813 (*SCC1-Myc18*, *SCC3-HA6*), 1625 (*SCC1-Myc18*, *SCC3-HA6*-degron), 10589 (*SCC1-MYC18*) and 1906 (*SCC1-MYC18, Δwpl1*) were staged in G1 with *α*-factor and released into media with nocodazole. Western blot was probed with anti-HA, anti-Myc and anti-Cdc28 antibodies. The same yeast cultures were used for chromosomal spreads (Figure 5) and for FACS analysis of cellular DNA content (Figure S13A). (D and E) Determination of Scc3 and Pds5 copy number per yeast cell. (D) Coomassie-stained gel with serial dilutions of purified recombinant Scc3-HA6, Pds5-HA6 and BSA (NEB #B9001) as a standard. (E) Protein extracts were prepared from nocodazole-arrested strains 1323 (*SCC3-HA6*-degron), 1479 (*SCC3-HA6*), 1675 (*PDS5-HA6*-degron) and 1677 (*PDS5-HA6*). Extracts from the indicated number of cells were analyzed by Western blot with anti-HA antibody. Known quantities of recombinant Scc3-HA6 and Pds5-HA6 were used as standards. Bands were quantified with MetaMorph.

In experiments employing a Scc1-Eco1 fusion construct, we discovered that the fusion protein was destroyed in cells arrested in G2 by nocodazole treatment (Figure S3A), which resulted in lethality unless an “unfused” wild type version of Scc1 was co-expressed. This observation led us to examine the abundance of the Eco1 protein throughout the cell cycle. We found that Eco1 is down-regulated when cells exit S-phase and is dramatically reduced in G2 arrested cells (Figure 3A). During the preparation of this manuscript, the existence of a Cdk1-dependent degron was reported for Eco 1 [35].

Surprisingly, and in contrast to the *SCC1-ECO1* strain, a *SCC3-ECO1* strain was viable when endogenous *SCC3* and *ECO1* genes were both deleted, indicating that the fusion protein can fulfill the essential functions of both Scc3 and Eco1. Nevertheless, the Scc3-Eco1 fusion protein was destroyed in nocodazole arrested cells similarly to the Scc1-Eco1 fusion (Figure S3C).

The Eco1 protein is comprised of three domains: an N-terminal region containing the PCNA-interacting PIP box and C_2_H_2_ Zinc-finger, the presumably unstructured S/P-rich middle region and the C-terminal acetyltransferase domain (Figure S3B). In order to determine the localization of degron sequences within Eco1 we fused *SCC3* and *SCC1* genes to each of the three domains of *ECO1*. Fusion of the N-terminal domain had no effect on the abundance of the fusion proteins while fusion of either the middle region or the acetyltransferase domain led to reduced protein levels throughout the cell cycle and disappearance of the fusion proteins after S phase (Figure 3B and Figure S3C).

In further experiments we exploited the ability of the middle region of Eco1 to induce protein degradation in order to address the function of Scc3 and Pds5. Endogenous *SCC3* and *PDS5* genes were tagged at their C-terminus with an HA6 sequence followed by amino acids 63–109 from the middle region of *ECO1*, hereafter referred to as “degron”. This system allowed us to monitor the abundance of Scc3 and Pds5 proteins throughout the cell cycle (Figure 3B). Fusion of *SCC3* and *PDS5* to a degron resulted in a dramatic reduction of the native levels of the respective proteins. However, the decrease in protein abundance was observed throughout the cell cycle. These results suggest that when fused to other proteins, the Eco1-derived degron does not necessarily have an ability to specifically induce protein degradation after the completion of S phase but rather reduces protein stability throughout the cell cycle. Importantly for our study, the amount of Scc1 reduced only slightly in the *PDS5* degron strain and even modestly elevated in the *SCC3* degron strain (Figure 3C). With the use of N-terminal Myc tag in addition to C-terminal HA tag and degron we were able to confirm that degradation of Scc3 and Pds5 proceeded to completion and no stable fragments could be detected in the degron fusion strains (Figure S3D).

We estimated the absolute numbers of Scc3 and Pds5 molecules per cell remaining in degron strains. Serial dilutions of highly purified recombinant Scc3-HA6 and Pds5-HA6 proteins were compared to dilutions of cell lysates from the wild type and degron strains on a Western blot (Figure 3D and E). This analysis allows us to estimate that in nocodazole arrested budding yeast there are approximately 4500 molecules of Scc3 and 10000 molecules of Pds5 per cell, which is in agreement with the numbers provided in the yeast database (www.yeastgenome.org). In the respective degron strains, there are approximately 250 molecules of Scc3 and Pds5 each, or about 15 molecules per chromosome assuming that 100% of Scc3 and Pds5 are associated with cohesin complexes and loaded on the DNA which is most likely an overestimate. A similar analysis performed with Scc1 resulted in an estimate of 4000 Scc1 molecules per haploid yeast genome [36]. Therefore in the degron strains only 6% or less of cohesin complexes can be associated with Scc3 or Pds5.

To confirm that Scc3 and Pds5 levels decreased due to protein turnover in degron strains, we incubated nocodazole-arrested cultures with cyclohexamide, an inhibitor of protein synthesis. As expected, the levels of wild type Scc3 and Pds5 as well as those of the Scc1 and Smc3 cohesin subunits remained stable throughout the 120 minutes incubation. In contrast, the abundance of the Scc3-degron and Pds5-degron proteins was significantly reduced upon inhibition of protein synthesis (Figure S4).

### Pds5 and Scc3 recruit Wpl1 to cohesin complexes

**Figure 4:**
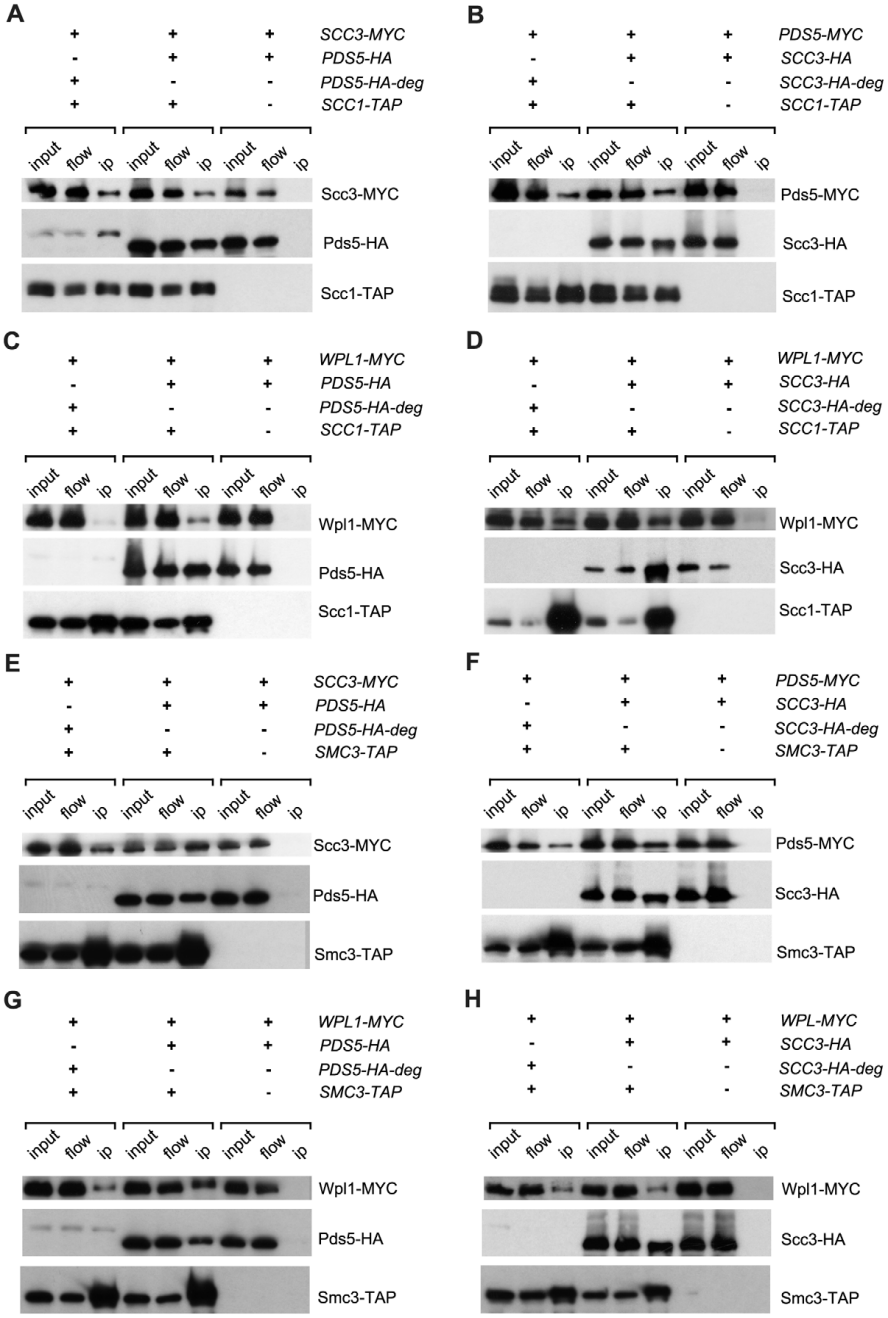
Interaction of Pds5, Scc3 and Wpl1 with cohesin ring. Lysates of nocodazole/benomyl arrested yeast cultures were incubated with IgG sepharose to precipitate Scc1-TAP or Smc3-TAP. The presence of different proteins on the IgG beads was analysed by Western blot probed with anti-HA (12CA5), anti-MYC (71D10) and PAP (P1291, Sigma). The strains were in (A): 1771 (*SCC3-MYC18, PDS5-HA6*), 1829 (*SCC3-MYC18, PDS5-HA6*-degron, *SCC1-TAP*), 1958 (*SCC3-MYC18, PDS5-HA6, SCC1-TAP*); in (B): 1734 (*PDS5-MYC18, SCC3-HA6*), 1834 (*PDS5-MYC18, SCC3-HA6*-degron, *SCC1-TAP*), 1956 (*PDS5-MYC18, SCC3-HA6, SCC1-TAP*); in (C): 1882 (*WPL1-MYC18, PDS5-HA6*), 2014 (*WPL1-MYC18, PDS5-HA6, SCC1-TAP*), 2016 (*WPL1-MYC18, PDS5-HA6*-degron, *SCC1-TAP*); in (D): 1880 (*WPL1-MYC18, SCC3-HA6*), 2012 (*WPL1-MYC18, SCC3-HA6, SCC1-TAP*), 2018 (*WPL1-MYC18, SCC3-HA6*-degron, *SCC1-TAP*); in (E): 1771 (*SCC3-MYC18, PDS5-HA6*), 2251 (*SCC3-MYC18, PDS5-HA6, SMC3-TAP*), 2290 (*SCC3-MYC18, PDS5-HA6*-degron, *SMC3-TAP*); in (F): 1734 (*PDS5-MYC18, SCC3-HA6*), 2249 (*PDS5-MYC18, SCC3-HA6, SMC3-TAP*), 2264 (*PDS5-MYC18, SCC3-HA6*-degron, *SMC3-TAP*); in (G): 1882 (*WPL1-MYC18, PDS5-HA6*), 2253 (*WPL1-MYC18, PDS5-HA6, SMC3-TAP*), 2265 (*WPL1-MYC18, PDS5-HA6*-degron, *SMC3-TAP*); in (H): 1882 (*WPL1-MYC18, PDS5-HA6*), 2261 (*WPL1-MYC18, SCC3-HA6, SMC3-TAP*), 2271 (*WPL1-MYC18, SCC3-HA6*-degron, *SMC3-TAP*).

To determine the effects of Scc3 and Pds5 depletion on the architecture of cohesin complexes, we performed the immunoprecipitation experiments (Figure 4). Scc3 could be efficiently co-immunoprecipitated with Scc1 or Smc3 in *PDS5*-degron strains and Pds5 could be co-immunoprecipitated with Scc1 and Smc3 in *SCC3*-degron strains, indicating that Scc3 and Pds5 associate with the cohesin ring independently of each other.

In order to determine whether binding of Wpl1 to cohesin rings requires Scc3 or Pds5, we immunoprecipitated Scc1 (Figure 4C and D) and Smc3 (Figure 4G and H) from wild type, *SCC3*-degron or *PDS5*-degron strains and detected Wpl1 in the pull-down fraction by Western blot. Wpl1 was co-immunoprecipitated with “core” cohesin subunits in the wild type strain. However, the amount of cohesin-bound Wpl1 was reduced in the absence of Pds5 indicating that Pds5 is important for Wpl1 recruitment to cohesin. Depletion of Scc3 resulted in a less pronounced reduction of cohesin-associated Wpl1 suggesting a minor role of Scc3 in Wpl1 recruitment.

### Scc3 and Pds5 are not required for the maintenance of the bulk of cohesin on DNA

**Figure 5:**
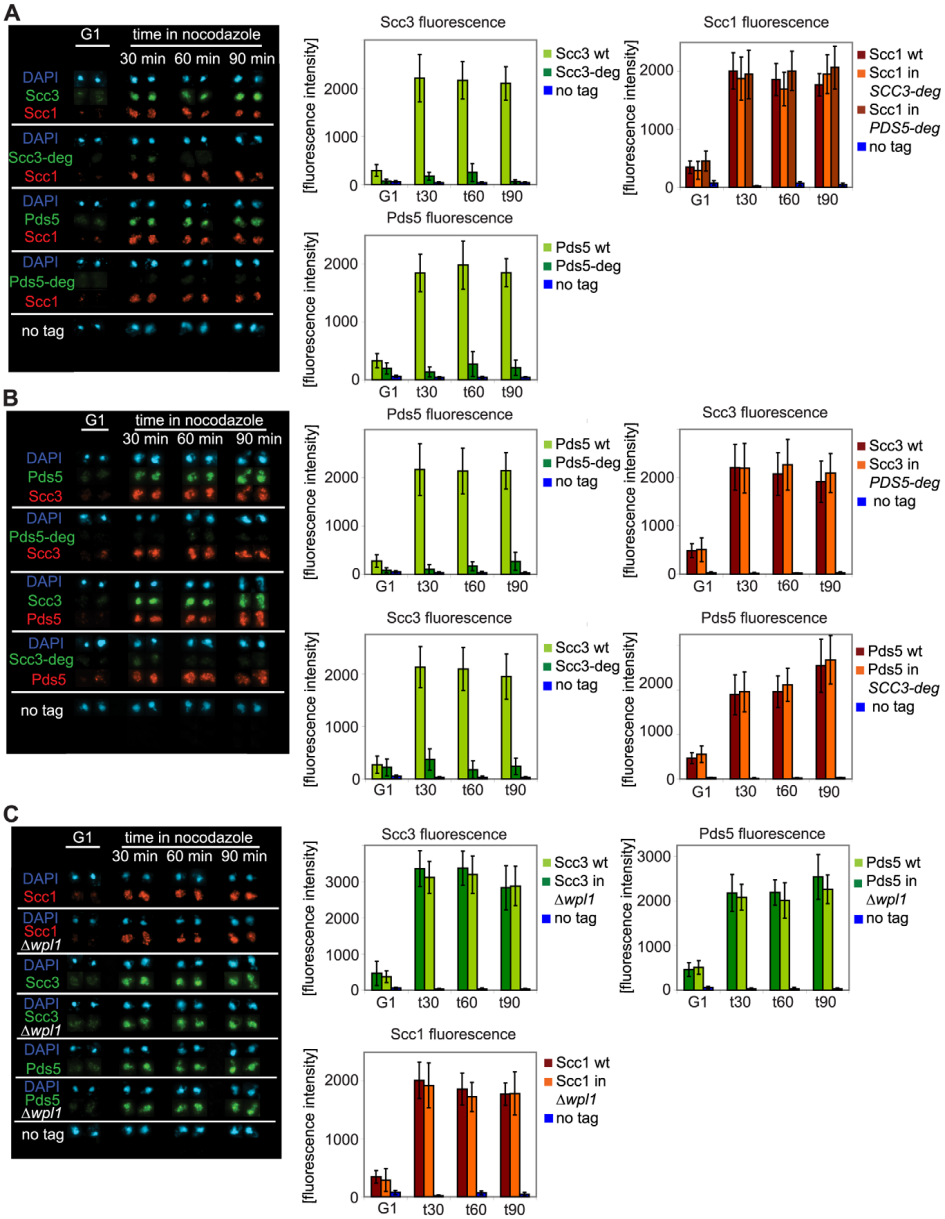
Depletion of Scc3 and Pds5 does not affect cohesin association with chromatin. Yeast strains were staged in G1 with *α*-factor and released into media with nocodazole. Chromosomal spreads were prepared as in Figure 2. At every time point fluorescence of 50 nuclei was determined. Error bars represent standard deviation. FACS analysis of cellular DNA content is shown in Figure S13. The strains were in (A): 1813 (*SCC1-Myc18*, *SCC3-HA6*), 1625 (*SCC1-Myc18*, *SCC3-HA6*-degron), 1815 (*SCC1-Myc18*, *PDS5-HA6*), 1818 (*SCC1-Myc18*, *PDS5-HA6*-degron), in (B): 1771 (*SCC3-Myc18*, *PDS5-HA6*), 1796 (*SCC3-Myc18*, *PDS5-HA6*-degron), 1734 (*PDS5-MYC18*, *SCC3-HA6*) and 1744 (*PDS5-Myc18*, *SCC3-HA6*-degron) in (C): 1479 (*SCC3-HA6*), 1864 (*SCC3-HA6, Δwpl1*), 1677 (*PDS5-HA6*), 1866 (*PDS5-HA6, Δwpl1*), 10589 (*SCC1-Myc18*) and 1906 (*SCC1-Myc18, Δwpl1*).

To confirm that most of the cohesin complexes loaded onto chromosomes in the degron strains are devoid of Scc3 and Pds5, we compared the amounts of Scc3 and Pds5 wild type and degron-fused proteins in chromosome spreads (Figure 5A) and in chromatin pellets (Figure S5). While wild type Scc3 and Pds5 were readily detectable on chromosomes during S phase and G2, the fusion proteins were non-detectable throughout the cell cycle. Remarkably, a dramatic reduction in the amounts of Scc3 and Pds5 bound to chromosomes in the degron-fusion strains did not result in the reduction of chromosome-associated Scc1 (Figure 5A and Figure S5) or Smc3 (Figure S6). We conclude that stoichiometric quantities of Scc3 and Pds5 are not required to maintain cohesin on DNA. In addition, we could not detect any reduction in the amount of Pds5 bound to chromosomes in the *SCC3*-degron strain or any reduction in the amount of Scc3 bound to chromosomes in the *PDS5*-degron strain (Figure 5B), suggesting that they bind to DNA independently of each other. In order to address whether Scc3 and Pds5 can bind to chromatin independently of cohesin rings we performed chromosome spreads as the cells were released from G1 arrest in the absence of Scc1 (Figure S7). A yeast strain expressing Scc1 from the GAL promoter was arrested in G1 with α-factor and then released from the arrest into media containing galactose or glucose. No cohesin was detected on chromatin during G1 arrest. In galactose the appearance of full length Scc1 correlated with Scc1, Scc3 and Pds5 being detected on chromosome spreads while in glucose-containing media the level of Scc1 remained very low and no Scc3 and Pds5 were detected on chromosomes. Therefore, most if not all Scc3 and Pds5 are recruited to chromosomes by cohesin rings.

As Scc3 and Pds5 were both reported to associate with Wpl1, we examined whether Wpl1 affects the association of Scc3 and Pds5 with chromosomes. We could not detect any change in the amounts of Scc3, Pds5, Smc3 or Scc1 on chromosomes in the *Δwpl1* strain compared to wild type (Figure 5C and Figure S6). We were not able to detect Wpl1 in chromosome spreads under our experimental conditions which precluded the reciprocal experiment.

Since fluorescence measurements performed on chromosomal spreads provide only crude estimates of the quantity of cohesin associated with chromosomes, we performed ChIP-qPCR analysis on the Scc1 associated with the centromere-adjacent region, or with cohesin positive or negative sites on chromosomal arms (Figure S8). The Scc1 ChIP was normalized to the efficiency of histone H3 IP at the same loci as described in [15]. Using this careful analysis we could detect a marginal decrease in the amount of Scc1 associated with the centromere-adjacent region or chromosomal arm sites in the *SCC3*-degron strain compared to wild type. Consistent with a previous report [15], we observed a 2–3 fold reduction in the amount of Scc1 bound to chromatin in the *Δwpl1* and *PDS5*-degron strains. Remarkably, *wpl1* deletion has a very similar effect on the amount of Scc1 associated with chromosomal loci as does the depletion of Pds5, although *WPL1* is non-essential and its deletion results in a relatively small sister chromatid cohesion defect (see below).

**Figure 6:**
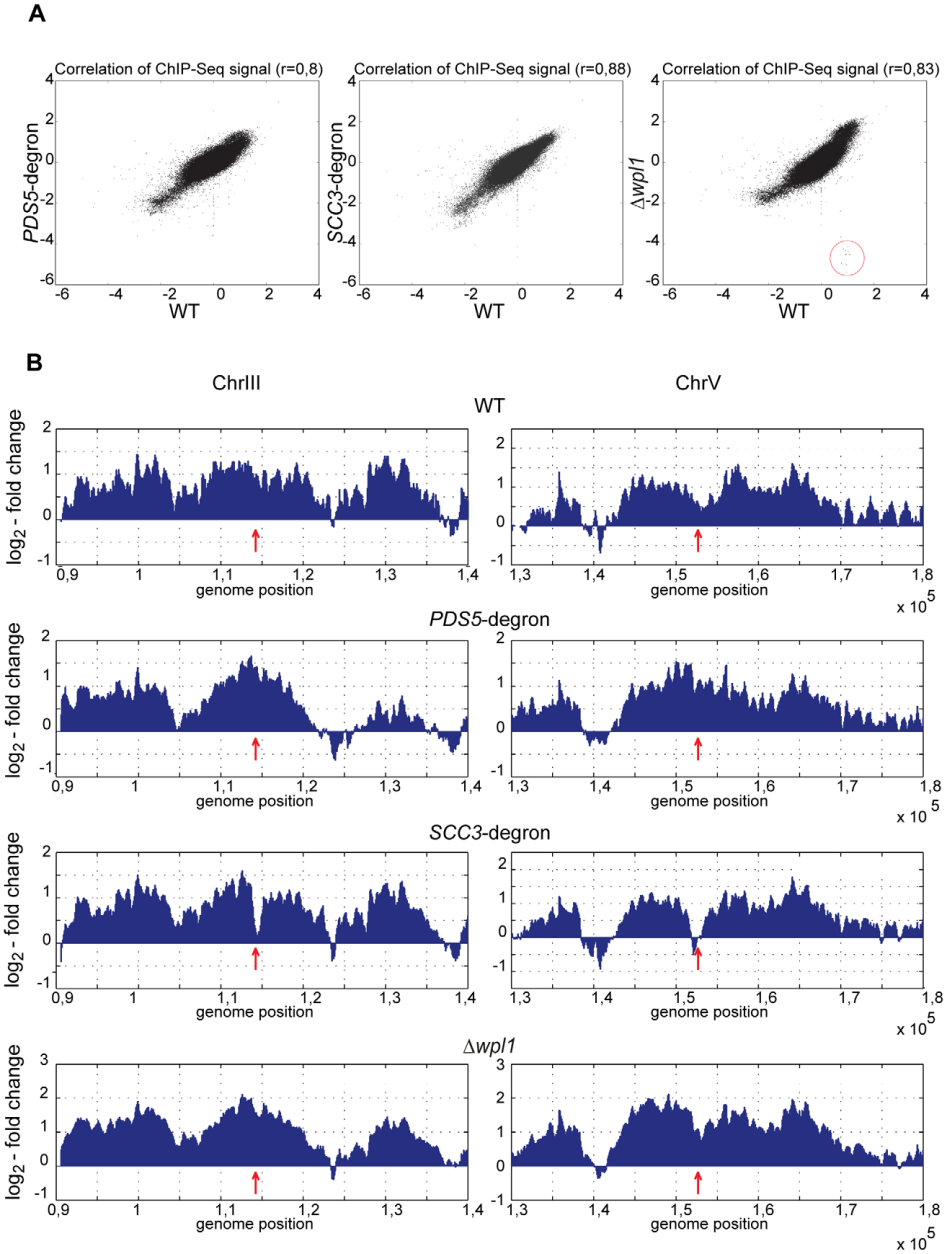
Genome-wide distribution of cohesin in the strains depleted of Scc3, Pds5, and Wpl1. Scc1-Myc18 ChIP was performed with nocodazole arrested 10589 (wild type, *SCC1-Myc18*), 1625 (*SCC3-HA6*-degron, *SCC1-Myc18*), 1818 (*PDS5-HA6*-degron, *SCC1-Myc18*), and 1906 (*Δwpl1, SCC1-Myc18*) strains. Untagged 1021 (wild type) strain was used as a control to determine signal log ratio. A running 500 bp window with a 50 bp step size was used to estimate local Scc1 abundance on chromosomal DNA. (A) Scatter plot between chromosomal Scc1 distributions in wild type vs *SCC3*-degron, *PDS5*-degron and *Δwpl1* strains. Regions corresponding to *WPL1* gene that are absent in *Δwpl1* strain are circled. (B) Scc1 distribution in the pericentromeric regions of chromosomes 3 and 5. Position of the core centromere is marked with an arrow.

To determine whether Scc3 or Pds5 target cohesin rings to distinct sites on chromosomes, we investigated the genome-wide distribution of cohesin in wild type, *SCC3*-degron, *PDS5*-degron and *Δwpl* strains arrested in G2 with nocodazole. Using a ChIP-Seq approach, we found that the overall Scc1 distribution was very similar in *SCC3*-degron, *PDS5*-degron and *Δwpl* strains compared to wild type with correlation coefficients 0.88, 0.80 and 0.83, respectively (Figure 6A and Figure S9A). We also could not detect any reproducible differences in cohesin distribution around the centromeres (Figure 6B) and at the tDNA genes (Figure S9B) which serve as the sites of cohesin loading onto DNA and are associated with cohesin loader Scc2/Scc4 complex [30].

### Scc3 and Pds5 are not required for the stable association of cohesin with DNA

**Figure 7:**
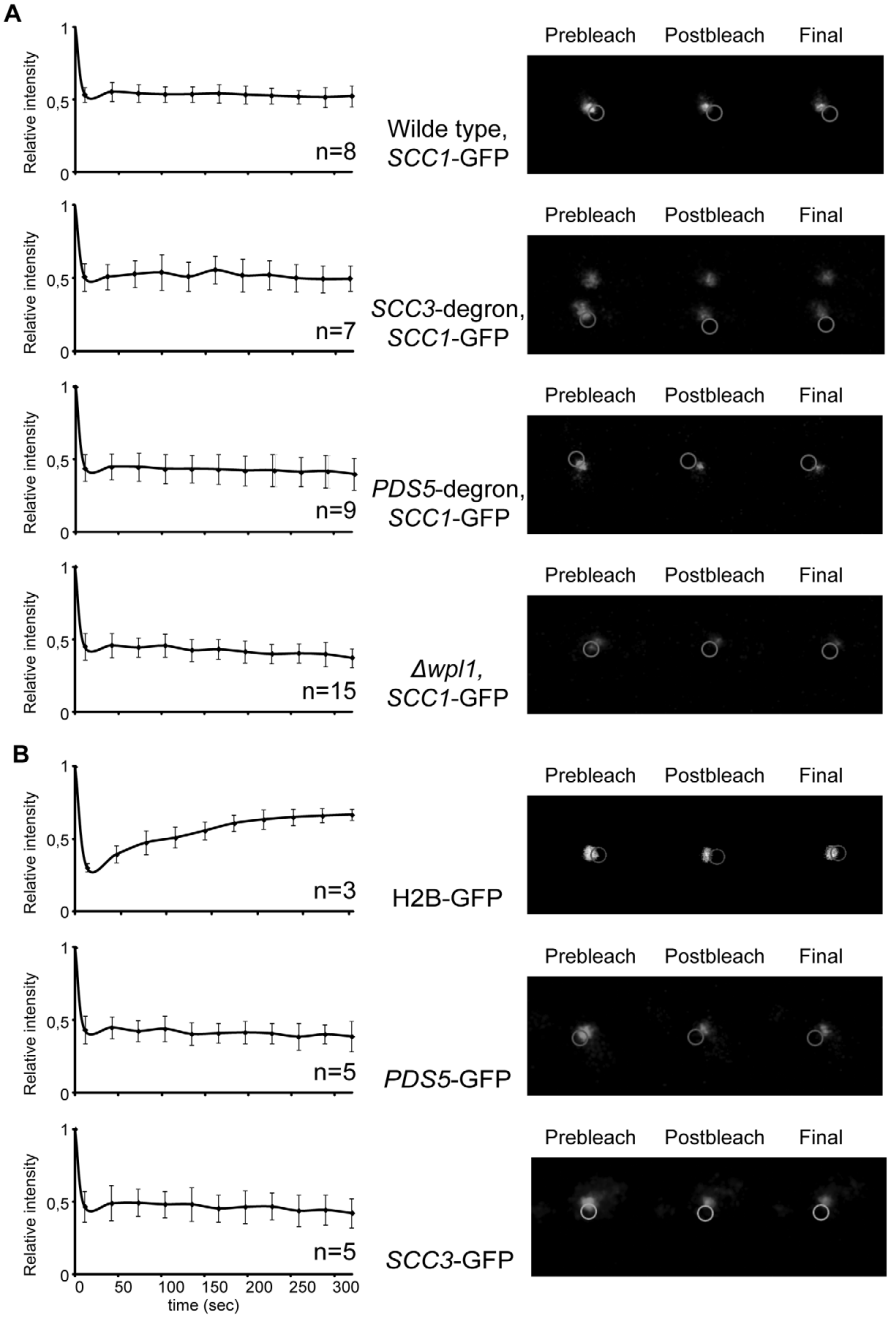
Depletion of Scc3, Pds5, and Wpl1 does not affect cohesin turnover rate on chromosomes. (A) A fluorescence recovery after photobleaching experiment was performed on mitotic cells of 2353 (wild type), 2390 (*SCC3-HA6*-degron), 2389 (*PDS5*-*HA6*-degron), and 2391 (*Δwpl1*) strains with endogenous *SCC1* tagged with GFP. No recovery of the bleached pericentric cohesin was observed during the experiment. (B) Scc3 and Pds5 stably associate with chromatin. A FRAP experiment was performed on mitotic cells of 2281 (*SCC3*-GFP) and 2417 (*PDS5*-GFP) strains. Histone H2B-GFP strain (1904) was used as control. No recovery of bleached Scc3-GFP and Pds5-GFP was observed during experiment in contrast with H2B-GFP. The mean and standard deviation were calculated from independent experiments (numbers of observed cells for each strain are indicated on the graphs).

Although the amount of cohesin on chromosomes in *SCC3*-degron and *PDS5*-degron strains was similar to the wild type strain, it remained possible that the association of cohesin rings with DNA is less stable when Scc3 or Pds5 are missing. To test this possibility we performed a fluorescence recovery after photobleaching (FRAP) experiment. We used yeast strains in which endogenous *SCC1* (Figure 7A) or *SMC3* (Figure S10) genes were tagged with *GFP*. It was previously reported that, in metaphase cells, Smc3-GFP is concentrated between the separated spindle poles and forms a cylindrical array where it is stably bound to DNA. It does not recover fluorescence after photobleaching [18], [37]. In contrast, an ATP hydrolysis-defective Smc3-GFP mutant that is unstably bound to centromeres, forms distinct foci in the nucleus instead of the cylindrical “barrel” and rapidly recovers fluorescence, t_1/2_ = 3.4 s [30]. In our experiments we photobleached a portion of the GFP fluorescence in metaphase cells and did not observe fluorescence recovery for the duration of the experiment (5 minutes) in either the wild type, *SCC3*-degron, *PDS5*-degron or *Δwpl1* strains. Histone H2B-GFP was used as a control and recovered fluorescence in parallel experiments (Figure 7B). Thus, cohesin rings are capable of maintaining a stable association with DNA in vivo in the absence of Wpl1 and when the amounts of Scc3 and Pds5 are greatly reduced. We conclude that the essential function of Pds5 and Scc3 cannot be the maintenance of cohesin rings in the closed state when on DNA.

As Pds5 associates with cohesin rings in a salt-sensitive manner and is present in sub-stoichiometric amounts in cohesin immunoprecipitates from cells, we used FRAP to determine whether there is significant turn-over of Pds5 and Scc3 on chromosomes. We did not observe any fluorescence recovery of Pds5-GFP and Scc3-GFP in our experiments (Figure 7B). Therefore, Pds5 and Scc3 are likely to be stable subunits of DNA-bound cohesin complexes in the cell under physiological conditions. Wpl1-GFP does not form a cylindrical array in metaphase cells and thus could not be photobleached. The diffuse fluorescent pattern observed for Wpl1-GFP suggests that it is not a stable cohesin subunit in the cell or that it is associated with only a small fraction of the cohesin complexes.

**Figure 8:**
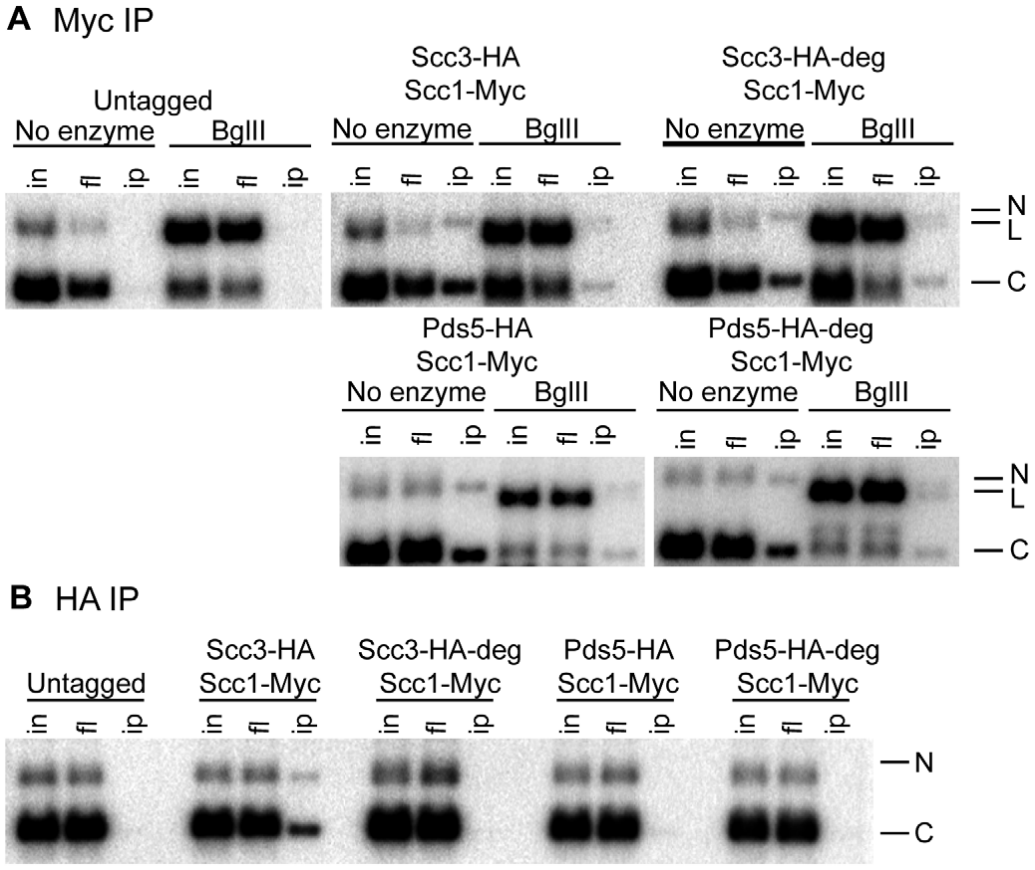
Cohesin rings devoid of Scc3 and Pds5 topologically embrace circular DNA. Strains 1021 (untagged), 1813 (*SCC3-HA6*, *SCC1-Myc18*), 1625 (*SCC3-HA6-*degron, *SCC1-Myc18*), 2525 (*PDS5-HA6*, *SCC1-Myc18*) and 1818 (*PDS5-HA6*-degron, *SCC1-Myc18*) carried the centromeric minichromosomes. (A) Yeast lysates were incubated with BglII restriction enzyme as indicated. Minichromosomes were co-immunoprecipitated with Scc1-Myc18. DNA was prepared by phenol/chloroform extraction and separated on a 1% agarose gel with ethidium bromide. Southern blot probed with a *TRP1*-specific probe is shown. Nicked (N), linear (L), and closed circular (C) forms of the minichromosome are indicated. (B) Minichromosomes were immunoprecipitated with anti-HA antibody. Minichromosomes from *SCC3-HA6* but not *SCC3-HA6*-degron strains could be co-immunoprecipitated with Scc3 indicating the efficient depletion of Scc3 from the minichromosomes in the *SCC3-HA6*-degron strain. Since Pds5 association with minichromosomes is very salt-sensitive, they could not be co-immunoprecipitated with Pds5-HA6 in either the wild type or *PDS5-HA6*-degron strains under our experimental conditions.

To demonstrate that cohesin rings devoid of Scc3 and Pds5 are topologically embracing DNA we employed a minichromosome-based assay that was described earlier [38]. Circular minichromosomes from the wild type, *SCC3*-degron and *PDS5*-degron strains could be co-immunoprecipitated with Scc1. However, the linearization of minichromosomes resulted in the dissociation of cohesin from the DNA due to sliding of the cohesin ring off the DNA end (Figure 8). We conclude that cohesin rings devoid of Scc3 or Pds5 maintain their topological association with the DNA.

### Scc3 and Pds5 are required for efficient sister chromatid cohesion

**Figure 9:**
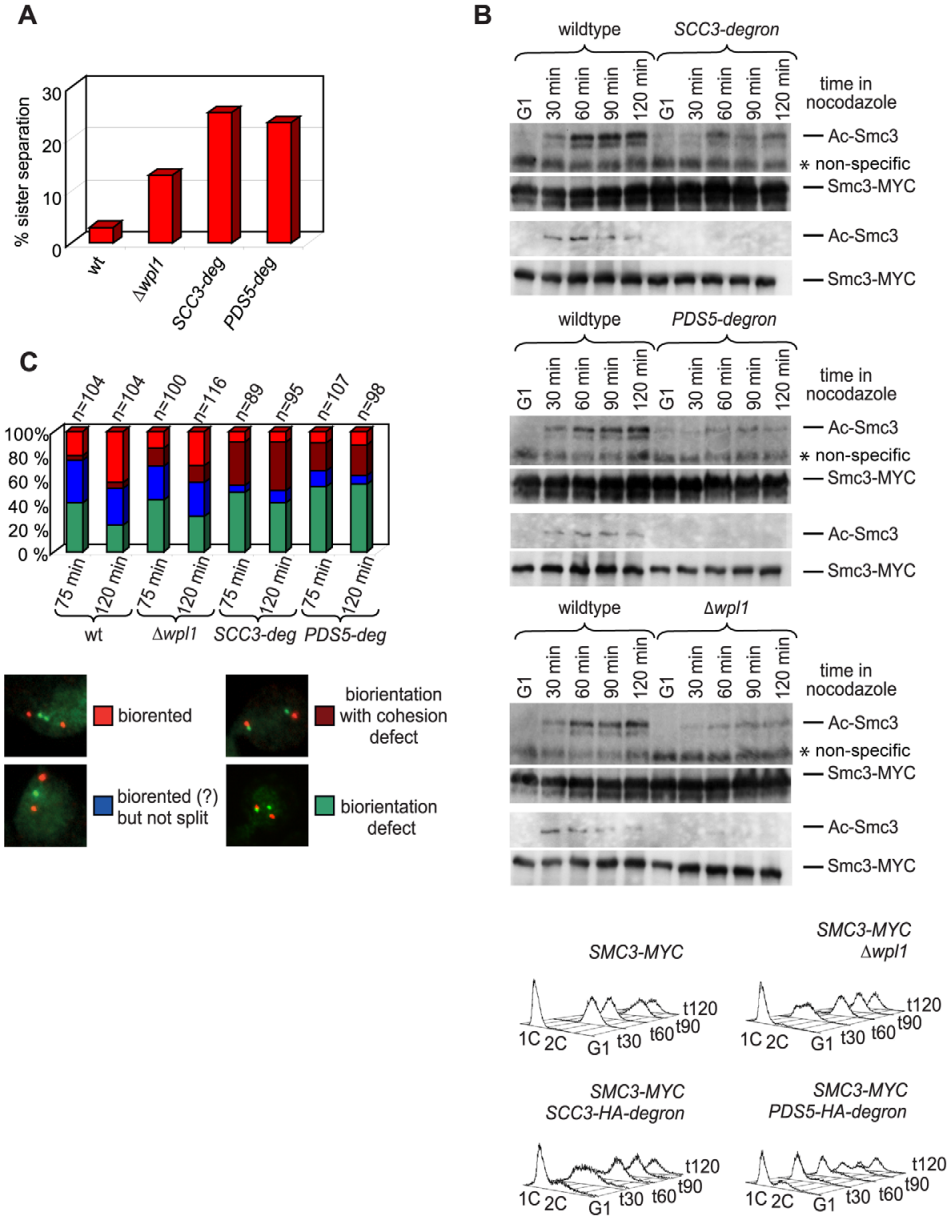
Sister chromatid cohesion defect in yeast depleted of Scc3 and Pds5. (A) Yeast strains 1417 (wild type), 2190 (Δ*wpl1*), 1621 (*SCC3*-degron) and 1678 (*PDS5*-degron) have an array of 200 Tet operators integrated into *URA3* locus 35 kb from the centromere on chromosome V and express TetR-GFP. Cells were staged in G1 with *α*-factor and released into media with nocodazole for 100 minutes. Separation of sister chromatids was scored as one (non-separated) versus two (separated) GFP dots. Dots separation was scored in 300 cells per strain. (B) 1759 (wild type), 1776 (*SCC3*-degron), 1779 (*PDS5*-degron) and 1769 (*Δwpl1*) strains with Myc-tagged endogenous *SMC3* gene were released from *α*-factor arrest into nocodazole containing media. Samples were collected at the indicated time points and processed for Western blot probed with anti-acetyl-Smc3 antibody (see Figure S11). The same blot was re-probed with anti-MYC to detect Smc3 (71D10). Western blots with different amounts of samples are shown to demonstrate both the actual levels of Ac-Smc3 in different strains and equal loading. FACS analysis of cellular DNA content is shown. (C) 1822 (wild type), 2436 (Δ*wpl1*), 1832 (*SCC3*-degron) and 1833 (*PDS5*-degron) strains with CENIV GFP dots and Spc42-Tomato were expressing Cdc20 from methionine-repressible promoter. Strains were arrested in G1 with *α*-factor and methionine was then added for 1 hour to shut down Cdc20 expression. Cells were released from G1 arrest into methionine and nocodazole containing media for 2 hours. Microtubule poisons were washed out and samples taken at indicated times, fixed in methanol and analyzed by fluorescence microscopy.

Although we found no obvious defects in cohesin association with DNA, sister chromatid cohesion was significantly weakened in *SCC3*-degron and *PDS5*-degron strains (Figure 9A). Premature sister separation in the *Δwpl1* strain was less frequent than in the degron strains. In order to test whether the cohesion defect could be at least partially due to lack of Smc3 head acetylation we generated an antibody that specifically recognizes acetylated Smc3 (Figure S11) and examined the level of Smc3 acetylation in synchronized yeast cultures that were progressing from G1 into S phase. The acetylated form of Smc3 was readily detectable in S phase and was reduced in the *SCC3*-degron, *PDS5*-degron and *Δwpl1* strains when compared to wild type (Figure 9B). This reduction in Smc3 acetylation might contribute to the sister chromatid cohesion defect observed for these strains.

Deletion of *wpl1* was reported to make *ECO1* dispensable for viability in yeast [39]. To determine whether down-regulation of Scc3 or Pds5 alleviated the requirement of *ECO1*, we crossed *SCC3*-degron and *PDS5*-degron strains to a strain in which *eco1* deletion was rescued by a wild type *ECO1* transgene integrated at an unlinked ectopic locus. All of the resultant spores with the *eco1* deletion also carried a wild type *ECO1* transgene (38 spores in case of *PDS5*-degron and 26 spores in case of *SCC3*-degron) indicating that *ECO1* remains essential in *SCC3*-degron and *PDS5*-degron strains. It is possible that down-regulation of Scc3 and Pds5 weakened cohesion in the degron strains and that the Eco1 contribution to cohesion establishment became crucial even though Wpl1 could not efficiently associate with cohesin rings in these strains. We were unable to combine the *SCC3*-degron and *PDS5*-degron in one strain or to delete *WPL1* in either *SCC3*-degron or *PDS5*-degron strains.

Sister chromatid cohesion plays a key role in ensuring that sister kinetochores attach to opposite spindle poles (i.e. bi-orient) during cell division. The tug of war between microtubules pulling sister kinetochores to opposite spindle poles and cohesin rings resisting their splitting force generates tension across sister kinetochores. Only microtubule-kinetochore attachments that result in tension are stabilized, ensuring proper chromosomal segregation. To test whether the cohesion defect we observed resulted in the reduced ability of cells to bi-orient sister kinetochores in mitosis, we used a bi-orientation assay developed by [40]. Strains that carry an array of Tet operators integrated 2 kb from the centromere on chromosome IV and express a Tet repressor-GFP fusion are used to visualize kinetochores as fluorescent green dots. The spindle pole body component, Spc42, is tagged with tomato and can be detected as red dots. The anaphase promoting complex subunit, Cdc20, is expressed from a methionine-repressible promoter, which generates metaphase arrest in the presence of microtubules in methionine-containing media. The strains were arrested in G1 with α-factor and released into nocodazole and benomyl containing media. After cells arrested in G2 in the absence of microtubules, the drugs were washed out and cells were allowed to build mitotic spindles and establish kinetochore-microtubule attachments while remaining arrested in metaphase due to depletion of Cdc20. Split GFP dots aligned between red spindle pole bodies indicated that bi-orientation was established while two GFP dots at the same spindle pole or one dot at the pole and another at some distance from a pole signified a microtubule attachment defect (Figure 9C). *SCC3*-degron and *PDS5*-degron strains display an obvious sister chromatid cohesion defect and split kinetochores are frequently located at the spindle poles rather than being aligned in the middle of the spindle as in wild type cells. *SCC3*-degron and *PDS5*-degron strains were generally less efficient at establishing bi-orientation. However, about 50% of the cells in either of the degron strains do attach sister kinetochores to opposite spindle poles within 120 minutes of release from microtubule poison arrest indicating that sister chromatid cohesion is still sufficient to establish bi-orientation. As expected from its relatively small sister chromatid cohesion defect, the bi-orientation defect observed in the *Δwpl1* strain was less pronounced than in *SCC3*-degron and *PDS5*-degron strains.

While crossing *SCC3*-degron and *PDS5*-degron strains we noticed that they display an unusual phenotype. The *MAT α* versions of these strains readily mated with not only *MAT a*, but also with *MAT α* partners. The explanation of this “a-faker” phenotype could be the frequent loss of chromosome III carrying the mating type locus. *MAT α* cells that lose their *MAT* locus mate as if they are *MAT a*, the default state of budding yeast with a *MAT* deletion [41]. We confirmed that the illegitimate mating was indeed the result of the loss of chromosome III and not an epigenetic inactivation of the *MAT* locus or mating type switch. First, most of the resulting *α*/*α* diploids were unable to sporulate but mated with a *MAT a* tester strain indicating that there was no mating type switch. Second, when mating a degron strain with a TetR-GFP fusion integrated into *LEU2* locus on the opposite arm of chromosome III, no GFP expression could be detected in the illegitimate diploids. The frequency of *α* to *α* mating in the *SCC3*-degron strain was estimated to be about 200 times higher than for wild type (Figure S12A). Thus depletion of Scc3 and Pds5 results in elevated rates of chromosomal mis-segregation consistent with sister chromatid cohesion and bi-orientation defects.

*SCC3*-degron and *PDS5*-degron strains were extremely sensitive to X-ray irradiation (Figure S12B) while the *Δwpl1* strain demonstrated a rather modest increase in sensitivity and only at very high doses, in agreement with an earlier study [42]. As homologous recombination is the preferred pathway of double-strand break repair in post-replicative cells and depends on sister chromatid cohesion [43], it is possible that the observed sensitivity can be fully accounted for by weakened sister chromatid cohesion in these strains. It was reported that human cells depleted of cohesin accumulate spontaneous double-strand breaks because of defects in DNA damage repair [44]. We monitored the formation of Rad52-YFP foci [45] as *SCC3*-degron and *PDS5*-degron strains progressed through S phase. We did not detect any increase in the frequency of Rad52-YFP foci in these strains compared to wild type (data not shown). Therefore it appears that in the absence of radiation damage there is no dramatic increase in the DNA double strand breaks in Scc3 or Pds5-depleted strains indicative of an on-going repair process. In human cells cohesin, but not sister chromatid cohesion, is required for the activation of DNA damage-induced intra-S and G2/M checkpoints [44]. In budding yeast, a reduction in chromosomal cohesin resulted in hypersensitivity to DNA damage under conditions where sister chromatid cohesion was unaffected [46]. At this moment we cannot exclude the possibility that Scc3 and Pds5 themselves might have specific functions in double-strand break repair or checkpoint activation.

## Discussion

### Scc3 and Pds5 do not “lock” cohesin rings on DNA

Cohesin rings are composed of two Smc subunits, Smc1 and Smc3, which are locked together by the third subunit, Scc1. The integrity of all three subunits of the ring is a requirement for its stable association with DNA [38], [47]. The function of the additional proteins that associate with the ring, Scc3 and Pds5, appears to be much less clear. Both Scc3 and Pds5 are essential in *S. cerevisiae* and mutations in either results in sister chromatid cohesion defects. This in principle might indicate that these proteins also form an indispensable part of cohesin ring, e.g., prevent its spontaneous re-opening. In this study we tested the requirements of Scc3 and Pds5 for cohesin association with DNA.

Scc3 is stably associated with cohesin rings when they are purified from cells and yeast circular minichromosomes can be co-immunoprecipitated with Scc3 [38], indicating that it is bound to cohesin complexes both in solution and on DNA. In mammalian cells, the Scc3 orthologue SA1 was reported to be bound to DNA with a half-life similar to that of Scc1 [48]. In these experiments both Scc3 and Scc1 were dynamically bound to DNA prior to S phase. After S phase a more stably bound fraction was detected that was implicated in holding sister chromatids together. This would indicate that under normal circumstances there is little exchange of Scc3 subunits between cohesin rings. Pds5, on another hand, was reported to associate with cohesin rings less stably since it readily dissociates from them under elevated salt conditions in vitro [21]. Yeast circular minichromosomes could not be co-immunoprecipitated with Pds5 under conditions which allowed their co-immunoprecipitation with Scc1, Scc3 and Smc1 ([38] and Figure 8B). However, in our experiments no turn-over of Scc3 or Pds5 could be detected in the FRAP experiment in vivo, strongly suggesting that under physiological conditions in the cell both of these proteins are stably associated with chromosomal cohesin complexes. The diffuse fluorescence pattern observed for Wpl1-GFP, on another hand, indicates that Wpl1 is not stably bound to cohesins on DNA.

We investigated the consequences of destabilizing the Scc3 and Pds5 proteins and discovered that their total amounts in the cell can be greatly decreased without causing lethality. It was recently reported that a reduction in the cellular level of the Scc1 subunit of cohesin to 13% of wild type levels resulted in an obvious decrease in the amount of Scc1 detected on chromosomal spreads as well as in chromatin immunoprecipitation assays at chromosomal arm sites [46]. Remarkably, this reduction did not lead to defects in sister chromatid cohesion or chromosomal segregation indicating that sister chromatid cohesion can be accomplished by a much smaller number of cohesin complexes than are normally associated with yeast chromosomes. In contrast, the reduction of Scc3 and Pds5 levels in our experiments resulted in premature separation of sister chromatids and chromosomal mis-segregation, underscoring their crucial importance for cohesin function. At the same time, while Scc3 and Pds5 could not be detected by immunostaining on chromosome spreads, little or no decrease in the amount of Scc1 on chromosomes could be observed. Most of the chromosomal cohesin complexes in *SCC3*- and *PDS5*- degron strains are not associated with these proteins but remain stably bound to chromosomes at usual cohesin sites. The 50% reduction of chromosomal Scc1 detected in *PDS5*-degron strain with ChIP-qPCR approach can possibly be attributed to the less efficient recruitment of Wpl1 to cohesin complexes in this strain since *wpl1* deletion caused similar if not more pronounced effect.

Because our Eco1-derived degron is non-inducible, it does not allow us to specifically eliminate Scc3 or Pds5 in G2 of the cell cycle once cohesion has been established. Such an approach would allow us to test whether these proteins are required for the maintenance of sister chromatid cohesion, i.e., the capacity of the cohesin complex to hold sisters over a period of time once both sisters are captured. When we performed this experiment with a “conventional” inducible degron, destruction of Pds5 in G2 arrested cells had no obvious effect on cohesion or cohesin association with DNA. Destruction of Scc3 was accompanied by the destabilization of Scc1 and cohesin loss from chromosomes while the effect on sister chromatid cohesion was very modest. This result is in agreement with a previous study which demonstrated that an 8 fold decrease in the abundance of chromosomal Scc1 does not result in premature sister separation [46]. Overall, experiments performed using the “conventional” degron are consistent with our conclusion that Scc3 and Pds5 are not functioning as a cohesin lock on DNA.

It is unlikely that Scc3 and Pds5 act redundantly, that is, that they both contribute to the maintenance of cohesin rings on DNA but either one is sufficient. Although we were not able to combine the Eco1-derived *SCC3* and *PDS5*-degrons in one strain, in experiments with “conventional” degron depletion of both Scc3 and Pds5 closely mimicked the effect of Scc3 depletion alone, which was likely due to the induction of Scc1 degradation. Depletion of Pds5 alone using the “conventional” degron had little or no effect on cohesin association with DNA.

**Figure 10:**
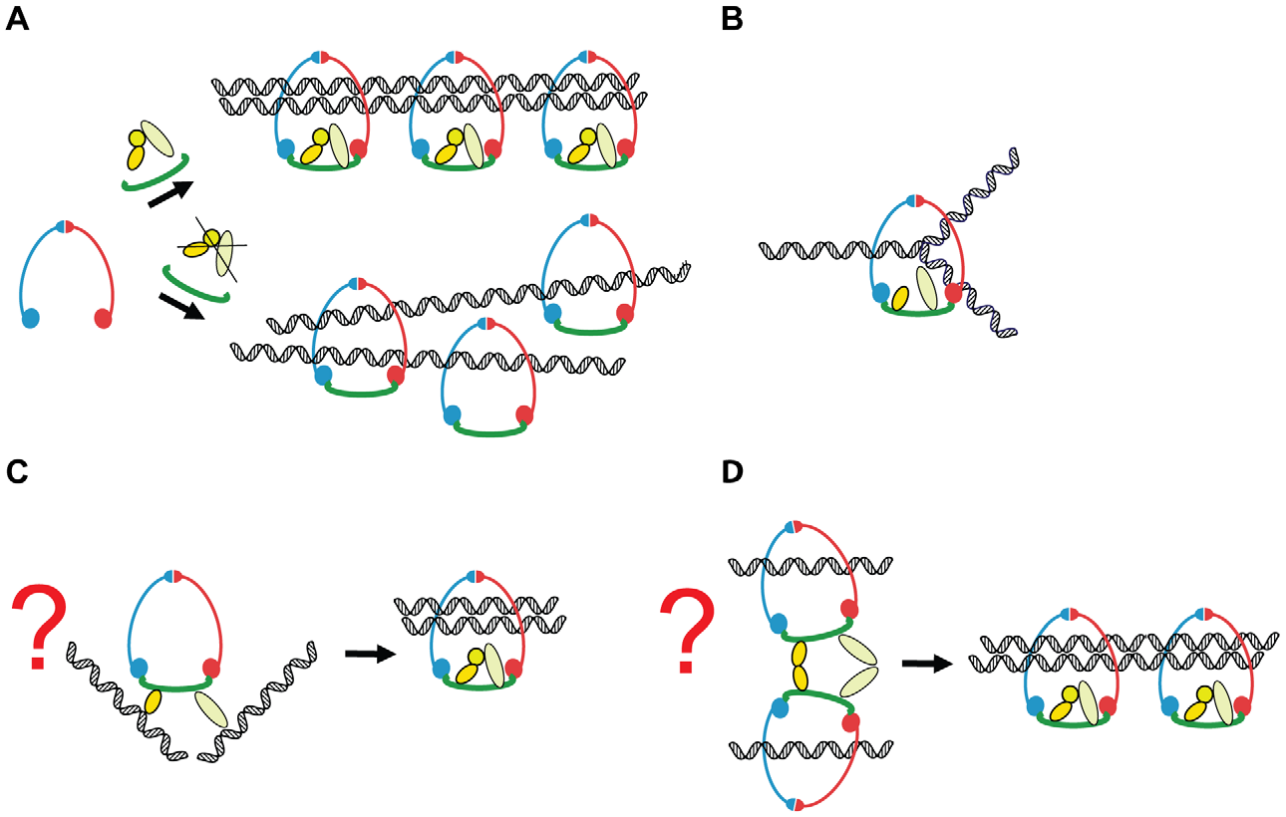
A model of how Scc3 and Pds5 play a role in the establishment of sister chromatid cohesion but are not required to stabilize cohesin rings on the DNA. In the normal cell cycle of budding yeast Scc1 subunit is synthesized in the late G1 or early S phase. Scc1 binds to Smc1/Smc3 heterodimer and completes the cohesin ring. Scc3 and Pds5 stably associate with cohesin via Scc1 subunit. Cohesin complexes are loaded on the chromosomes. During DNA replication two newly generated sister chromatids are captured inside a single cohesin ring in a process which remains poorly understood. Scc3 and Pds5 function to ensure that two sister chromatids are captured inside a cohesin ring. In their absence, cohesin complexes are stably loaded on the DNA but fail to embrace both of the sister chromatids resulting in defective sister chromatid cohesion (A). We can speculate that Pds5 and Scc3 could stabilize cohesin rings specifically during the replication fork passage (B) or transiently bind sister chromatids during the establishment of cohesion (C). Alternatively they could mediate a transient interaction between two cohesin rings as proposed by [61] (D).

In our experiments, depletion of Scc3 and Pds5 produced a phenotype that is not dissimilar to that of an *eco1* mutant, i.e., a defect in sister chromatid cohesion without a major effect on cohesin association with DNA (Figure 10). This correlation implies a role for Scc3 and Pds5 in cohesion establishment in spite of their reported role as counteracting factors of Eco1. Additionally, the essential functions of Scc3 and Pds5 can be accomplished by a much smaller number of molecules than normally present in the cell. It is possible that only very small number of cohesin rings actually hold sister chromatids together in our degron strains and that those rings do contain Scc3 and Pds5 subunits while the bulk of cohesin is bound to chromosomes without embracing both of the sisters. In this case, the amount of “cohesive” cohesin in our degron strains is limited to less than 15 complexes per chromosome. Importantly, the majority of cohesin complexes in these strains are devoid of Scc3 or Pds5 but still able to maintain stable association with DNA. It is possible that Scc3 and Pds5 are required transiently. For example, they may only be essential during ring loading on DNA and/or cohesion establishment and become non-essential once both sisters have been trapped inside the ring. Intriguingly, no premature separation of sister chromatids or significant effects on the cell cycle were observed in *Drosophila* cells depleted of SA/Scc3 using dsRNAi, although the protein was barely detectable [49].

Scc3 was implicated in the recruitment of cohesin to heterochromatic regions in fission yeast [50] and possibly in recruitment of cohesin to CTCF sites in human cells [51]. We tested whether Scc3 functions in determining the location of cohesin sites on chromosomes in budding yeast that lack pericentric heterochromatin or CTCF. We found that depletion of Scc3, Pds5, or Wpl1 did not have any major effect on the chromosomal addresses of the cohesin complex.

### Scc3, Pds5, Wpl1 and the architecture of cohesin complex

The immunoprecipitation experiments conducted in this study demonstrate that Pds5 and Scc3 associate with cohesin rings independently of each other. Scc3 is known to be recruited to cohesin via its interaction with Scc1 while the interacting partner of Pds5 on cohesin rings remains to be confirmed. While recombinant human Pds5 can directly bind to Scc1 in vitro, this interaction is enhanced in the presence of Scc3 [29]. In yeast, Pds5 binds to the cohesin ring in a Scc1-dependent manner. However, an analysis of intra-cohesin interactions in live yeast cells using fluorescence resonance energy transfer (FRET) revealed an unexpected interaction between Pds5 and the Smc1 hinge at the opposite side of the cohesin ring [52]. Since Pds5-Wpl1 and the Smc3 head bind to the same region of Scc1, Pds5 might reinforce the Scc1-Smc3 interface helping to maintain the integrity of the ring. However, a covalent Smc3-Scc1 fusion did not rescue *pds5* deletion (data not shown). The Smc3-Scc1 fusion was previously reported to suppress the lethality of mutations in the Scc1 N-terminal domain that reduce its interaction with the Smc3 head [31]. Therefore, maintaining the integrity of the cohesin ring at the Smc3-Scc1 interface cannot be the essential function of Pds5.

Of all the cohesin associated proteins, Wpl1 associates with the ring in the least stable fashion and displays a complicated pattern of interactions with the cohesin subunits. Wpl1 association with the cohesin ring in human cells is dependent on Scc3 and Scc1 with which it forms a stable ternary complex [17]. At the same time Wpl1 and Pds5 form another stable subcomplex that only weakly binds to the cohesin ring [16]. To complicate the matters, the N-terminal half of the vertebrate Wpl1, which is primarily responsible for its binding to cohesin, and the central region of Scc1, which is implicated in the interaction of Scc1 with Scc3, Pds5 and Wpl1, are conserved only in vertebrates [29]. Nevertheless, recombinant yeast Scc3, Pds5 and Wpl1 were reported to form a stable trimeric complex [18]. In this study we confirmed that Scc3, Pds5 and Wpl1 form a complex, although it is very unstable. Interestingly, the Pds5-Wpl1 dimer binds to the N-terminal region of Scc1 while the Scc3-Wpl1 dimer binds to the C-terminal region. Thus, Wpl1 may physically connect Scc3 with Pds5 and enable the trimeric complex to effectively span the length of Scc1. The functional importance of this architecture remains to be addressed experimentally.

The results of our immunoprecipitation experiments from *SCC3*-degron and *PDS5*-degron strains are consistent with the model that in yeast Wpl1 is primarily recruited to the cohesin ring via Pds5. Very little Wpl1 could be co-purified with the cohesin ring in the absence of Pds5. This result is in agreement with an earlier report in which the amount of Wpl1 on chromatin was reduced in the *pds5-r10* mutant [15]. Importantly, although *ECO1* can be deleted in the *Δwpl1* mutant or in yeast carrying certain specific point mutations in the *PDS5* or *SCC3* genes [9], [15], [18], simple destabilization of Scc3 or Pds5 proteins is insufficient to permit Eco1-independent growth (this study). Intriguingly, none of the *Δeco1* suppressor mutations in the *SCC3* and *PDS5* genes affected their interaction with Wpl1 in an in vitro assay [18]. It appears that *scc3* and *pds5* suppressor mutations influence the functions of the respective proteins in a more subtle way and do not result in partial loss-of-function alleles as was proposed earlier [15]. Rather, it is possible that these are the separation-of-functions alleles that selectively allow Scc3 and Pds5 to function in the establishment of cohesion while making them resistant to Wpl1 action that converts them from establishment to anti-establishment factors.

Interestingly, the level of Smc3 head acetylation was reduced when Scc3, Pds5 or Wpl1 were depleted. This reduction in Smc3 acetylation is unlikely to be the only reason for the observed cohesion defect since the *Δwpl1* strain displays a similar reduction in acetylation but only a very modest cohesion defect. In a previous study, reduced Smc3 acetylation in a *Δwpl1* strain was attributed to a decrease in chromosomal cohesin [39]. However, in our *SCC3*-degron strain, the amount of Scc1 associated with chromosomes is virtually unchanged despite diminished Smc3 acetylation.

In conclusion, we discovered a new and very efficient degron sequence and were able to employ it to study the effect of destabilization of Scc3 and Pds5. Our results demonstrate that Scc3 and Pds5 are not required for the maintenance of cohesin on DNA but are important for sister chromatid cohesion.

## Materials and Methods

### Strains and plasmids

Yeast strains are listed in Table S1.

Plasmid pYM27 [53] which was used for GFP-tagging of *SMC3*, *SCC1*, *SCC3*, *PDS5*, and *WPL1* as well as strain YKL200 and plasmids pKL187 [32], [33] and pCM324 [34] that were used for the construction of “conventional” temperature-sensitive degron strains were obtained from EUROSCARF.

### Protein expression and purification

Codon optimized sequences (Genescript) encoding Scc3, Pds5 and Wpl1 were cloned into pET21a or pET28b (Novagen). Sequences encoding N-terminal (aa 1–168), middle (aa 169–337) and C-terminal (aa 338–566) regions of Scc1 were cloned into pGEX-2T [7].

Proteins were tagged C-terminally with a His6 tag and expressed in *E. coli* BL21 (DE3) RIL according to common auto-induction protocols [54]. Cells were harvested and resuspended in lysis buffer (20 mM HEPES-KOH (pH 7,5), 300 mM NaCl, 5% glycerol, 5 mM imidazole, 1 mM β-ME, 1 mM PMSF and 1× complete EDTA-free protease inhibitor mix (Roche)). Cells were lysed (French Press, 17 kpsi), the cleared supernatant was incubated with Ni-NTA agarose beads (Quiagen) for 2 h at 4°C. Beads were washed with lysis buffer containing 500 mM NaCl and 30 mM imidazole and proteins were eluted with lysis buffer containing 100 mM NaCl and 250 mM imidazole. Subsequently, proteins were loaded onto a 16/60 Superdex200 or a 16/60 Superdex75 size exclusion column equilibrated with GF buffer (20 mM HEPES-KOH (pH 7,5), 100 mM NaCl, 5% glycerol, 1 mM β-ME). Peak fractions were analysed by SDS-PAGE and Coomassie staining and stored at −80°C.

10–30% Glycerol density gradients were performed in 10 mM HEPES-KOH (pH 7,5), 75 mM NaCl, 0,25 mM EDTA and 1× complete EDTA-free protease inhibitor mix (Roche)). Proteins were mixed and preincubated for 1 h at 4°C, loaded on a preformed gradient and centrifugated at 38000 rpm for 38 h at 4°C using a SW40 Ti rotor (Beckman Coulter). 300 µl fractions (44 fractions total) were harvested using a Gradient Station (Biocomp).

### GST pull-down

GST and GST-fusions with Scc1 were purified as described [55]. For GST pull-downs beads were equilibrated with binding buffer (20 mM HEPES (pH 7.5), 100 mM NaCl, 5% glycerol and 5 mM 2-mercaptoethanol). Wpl1, Scc3 and Pds5 were mixed and pre-incubated for 30 minutes at 4°C. GST-beads were then added and incubated with rotation for 2 hours at 4°C. Beads were washed three times with binding buffer containing 0.5% NP-40 and eluted by boiling in SDS-PAGE loading buffer.

### Immunoprecipitations

Yeast strains were grown in 200 mls of YEPD and arrested with 15 µg/ml nocodazole and 10 µg/ml benomyl for 2 hours at 30°C. Cells were harvested and lysates were prepared by beating with glass beads in buffer I (50 mM HEPES-KOH (pH 7,3), 70 mM Potassium Acetate, 5 mM Magnesium Acetate, 10% glycerol, 0,1% Triton X-100, 1× complete EDTA-free protease inhibitor mix (Roche), 2 mM PMSF, 1× PhosSTOP phosphatase inhibitor mix (Roche), 5 µg/ml aprotinin, 5 µg/ml pepstatin, 0,5 mM DTT). Protein concentrations of the lysates were adjusted and they were incubated with IgG Sepharose 6 beads (Amersham) for 2 hours or overnight. Beads were washed two times with buffer I, once with buffer I/100 mM Potassium Acetate, once with buffer I/120 mM Potassium Acetate, once with buffer I/150 mM Potassium Acetate. For detection of Wpl1 association with cohesin higher salt concentration washes were omitted and beads were washed three times with buffer I and two times with buffer I/100 mM Potassium Acetate.

### Minichromosome immunoprecipitation

Minichromosome immunoprecipitation was performed as described in [38]. Yeast strains were transformed with the 2310 bp plasmid [56] containing an 850 bp long *CEN4* sequence from YCplac22 and *TRP1ARS1* sequence. Strains were grown overnight in synthetic medium without tryptophan at 30°C, were diluted into 300 ml YEPD at OD_600_ of 0.2 and grown till OD_600_ reached 0.65. Cells were arrested with 10 µg/ml nocodazole for 1.5 hour. Spheroplasting was carried out with lyticase (L-2524, Sigma). Spheroplasts were lysed in 4.5 ml of lysis buffer (25 mM Hepes/KOH [pH 8.0], 50 mM KCl, 10 mM MgSO_4_, 10 mM Na citrate, 25 mM Na sulfite, 0.25% TritonX-100, 1 mM PMSF, 3 mM DTT, and 1× complete EDTA-free inhibitors [Roche]) supplemented with 100 ng/ml RNase A (Fermentas). DNA digest was performed with 1000 units of Bgl II per 1 ml of lysate for 2 hours at 4°C. The reaction was stopped by the addition of 5 M NaCl to a final concentration of 200 mM. Minichromosomes were immunoprecipitated with 12.5 µg/ml of anti-HA (12CA5, Roche) or anti-Myc (9E11, Santa Cruz) antibodies and 0.25 ml suspension of protein A dynabeads (Invitrogen). Minichromosomes were then eluted off the beads two times with 0.25 ml of 50 mM Tris (pH 8.0), 10 mM EDTA, 1% SDS at 65°C, extracted with phenol/chloroform/isoamyl alcohol (25∶24∶1) and ethanol precipitated. Samples were dissolved in 40 µg of TE and separated on a 1% agarose gel with ethidium bromide. Southern transfer was performed under denaturing conditions using Hybond-XL membrane (GE Healthcare). Blots were hybridized with a *TRP1* probe and scanned on Storm 840 (Molecular Dynamics).

### FRAP

Fluorescence microscopy and photobleaching were performed according to the protocol described in [37] with modifications. Cells were immobilized in a slab of media supplemented with 10% low melting point agarose (NuSieve GTG Agarose, Lonza) and imaged at the room temperature (20°C) with Olympus fv1000 laser scan confocal microscope with standard eGFP band pass filter (500–550 nm) and argon laser (488 nm excitation line). Seven z sections were acquired in 750 nm steps and analyzed with Imaris software. The mean and the standard deviation of the acquired intensity were calculated using MatLAB (Mathworks Inc., Natick, MA).

### ChIP-Seq

Chromatin immunoprecipitation was performed as in [57] using strains with Myc-tagged Scc1 subunit of cohesin, anti-Myc 9E11 antibody and rat anti-mouse IgG2a Dynabeads M-450 (Dynal). Immunoprecipitated DNA was ligated to the adaptors, size fractionated (150–500 bp) and amplified using ChIPSeq DNA Sample Prep Kit (Illumina) according to the manufacturer's instructions. DNA libraries were analysed on High Sensitivity DNA Chips with Bioanalyzer 2100 (Agilent Technologies). Cluster generation and sequencing analysis were performed on Illumina Genome Analyzer II with the help of Standard Cluster Generation V4-GA II and 36 cycle Sequencing v4 kits (Illumina). Reads were aligned against the *Saccharomyces cerevisiae* reference genome using Palmapper v0.5 [58] reporting all alignments with at most three mismatches and one gap. To reduce the effect of possible contamination with human DNA, we also aligned the reads against the human genome and only considered reads uniquely mapping to the yeast genome. We then used the multimapper resolution tool (http://bioweb.me/mmr) to identify the best location of reads mapping to multiple locations. For further analysis we only considered reads mapping to the chromosomal DNA of *Saccharomyces cerevisiae*. The summary of counts of aligned reads is presented in Table S2. We then computed and plotted the log-ratios between the samples' read coverages and the negative control (untagged strain) at equi-spaced genomic locations using Matlab.

### ChIP-qPCR

ChIP DNA was quantified by quantitative PCR using LightCycler 480 SYBR Green I Master mix and LightCycler 480 (Roche) according to manufacturer's instructions. Primers, used for quantification of the centromeric region of chromosome VI and a cohesin site on the arm of chromosome VI (172 kb) were described in [30]. Primers for a cohesin-low site on the arm of chromosome V (141 kb) were described in [15]. Histone H3 ChIP was performed with an anti-H3 rabbit polyclonal antibody (Abcam, ab1791) and Protein A Dynabeads (Invitrogen).

### Chromosome spreading

Chromosome spreading was performed as described in [59]. Images were taken with an Axio Imager fluorescence microscope (Zeiss) with a 63× objective and scaled with MetaMorph image analysis software version 7.1.3.0 (Molecular Devices). All the images were taken with the same exposure time and scaled using the minimum and maximum intensities recorded in the given data set as lower and upper limits respectively. Z stacks (7–9 planes with 0.3 micron step) were acquired and projected on one plane by averaging. An area of fixed size was placed over the chromatin region and the average intensity was recorded. Background was measured outside the chromatin area and subtracted. The same nuclei were used for quantification of Scc1 and Scc3/Pds5 signals. Figures were assembled with the help of Adobe Photoshop software.

### Other techniques

Rabbit polyclonal anti-acetyl Smc3 antibody was raised against a peptide CRTVGLK(Ac)K(Ac)DDYQL and affinity purified (Eurogentec).

Chromatin pellets were prepared as described in [60].

## Supporting Information

Figure S1 An induction of the “conventional” N-terminal ts degron results in a complete degradation of Pds5 and Scc3. To test if any stable fragments were remaining after the degron induction *PDS5* and *SCC3* were tagged at the C-termini with HA6. Degrons were induced in nocodazole-arrested yeast as in Figure 2A–C. Strain numbers are indicated in brackets in the figure. Western blots probed with anti-HA (16B12) antibody and anti-Cdc28 (sc-28550, Santa Cruz) as a loading control are shown. No stable fragments could be detected.
(TIF)

Figure S2 Sister chromatid cohesion defect in yeast depleted of Scc3 and Pds5 using a “conventional” N-terminal ts degron. (A) Strains 2418 (wild type), 2419 (degron-*PDS5*), 2420 (degron-*SCC3*), and 2449 (degron-*PDS5* and degron-*SCC3*) have an array of Lac operators integrated into *URA3* locus 35 kb from the centromere on chromosome V and express LacI-GFP. To induce the degron in G1, strains were staged with *α*-factor in YEP raffinose. Cells were resuspended in YEP galactose containing *α*-factor and incubated for 45 minutes at 30°C to induce the expression of Ubr1. Cells were then shifted to 37°C in YEP galactose containing doxycycline and *α*-factor and incubated for additional 90 minutes to destroy Pds5 and/or Scc3. Cells were released from *α*-factor arrest into YEP galactose containing nocodazole and doxycycline at 25°C for 3 hours. To induce degron in G2/M, strains were synchronized with *α*-factor and released into nocodazole containing YEP raffinose medium for 2 hours. Cells were resuspended in YEP galactose containing nocodazole and incubated for 45 minutes at 30°C to induce the expression of Ubr1. Cells were then shifted to 37°C in YEP galactose containing doxycycline and nocodazole and incubated for 90 minutes to destroy Pds5 and/or Scc3. Cells were then chased in YEP galactose containing nocodazole and doxycycline at 25°C for 3 hours. This last incubation step was found necessary since the dots signal was weakened under the conditions of degron induction. Separation of sister chromatids was scored as one (non-separated) versus two (separated) GFP dots in 300 cells. (B) FACS analysis of cellular DNA content. (C) Western blot demonstrating the depletion of Pds5 and Scc3. TCA protein extracts were prepared at indicated time points. Blots were probed with anti-Myc antibody (71D10) and anti-Cdc28 (sc-28550, Santa Cruz) for loading control.
(TIF)

Figure S3 Eco1 contains degron sequences. (A) 1188 (*GAL-SCC1*), 1176 (*GAL-SCC1 SCC1-HA6*) and 1177 (*GAL-SCC1 SCC1-HA6-ECO1*) strains were synchronized in G1 with *α*-factor and then released into media with glucose and nocodazole for 90 minutes. Western blot was probed with anti-HA antibody (12CA5). (B) Schematic representation of Eco1 domains. N-terminal region with the PCNA-interacting PIP box and C2H2 Zinc finger, middle region rich in serines and prolines and C-terminal acetyltransferase domain are indicated. (C) *GAL-SCC3* strains carrying transgenes *SCC3-HA6* (1257), *SCC3-HA6-ECO1* (1258), *SCC3-HA6-ECO1(aa1–63)* (1259) and *SCC3-HA6-ECO1(aa111–281)* (1260) were staged in G1 with *α*-factor and then released into galactose-containing media with nocodazole. Western blot was probed with anti-HA antibody (12CA5) to detect Scc3 or Scc3-Eco1 fusions. Loading control was anti-Cdc28 sc-28550 (Santa Cruz). (D) No stable fragments of Pds5 and Scc3 can be detected in the degron strains. *PDS5* and *SCC3* were tagged at the N-termini with Myc9. Strains were arrested in G2/M with nocodazole for 3 hours and TCA protein extracts were prepared. Strain numbers are indicated in brackets in the figure. Western blots probed with anti-Myc (71D10) antibody and anti-Cdc28 (sc-28550, Santa Cruz) as a loading control are shown.
(TIF)

Figure S4 Protein stability assay in nocodazole arrested cells. Strains 1323 (*SCC3-HA6*-degron), 1479 (*SCC3-HA6*), 1675 (*PDS5-HA6*-degron), 1677 (*PDS5-HA6*), 1759 (*SMC3-MYC18*), and 10589 (*SCC1-HA6*) were arrested with nocodazole for 2 hours. Cycloheximide was added at a final concentration of 0.1 mg/ml, samples were taken at the indicated time points and TCA protein extracts prepared. Western blot was probed with anti-HA (16B12) or anti-MYC (71D10) and anti-Cdc28 (sc-28550, Santa Cruz) antibodies as a loading control.
(TIF)

Figure S5 Chromatin-bound fraction of cohesin is not affected by the depletion of Scc3 and Pds5. Strains 1813 (*SCC1-Myc18*, *SCC3-HA6*), 1625 (*SCC1-Myc18*, *SCC3-HA6*-degron), 2525 (*SCC1-Myc18*, *PDS5-HA6*), 1818 (*SCC1-Myc18*, *PDS5-HA6*-degron) and 1906 (*SCC1-Myc18, Δwpl1*) were arrested in G2/M with nocodazole. Whole cell extracts (WCE) were fractionated into soluble supernatant (sup) and chromatin pellet (pell). Equivalent amounts of protein samples were separated on SDS-PAGE. Western blot was probed with anti-HA (16B12), anti-Myc (71D10), as well as with anti-Cdc28 (sc-28550, Santa Cruz) and anti-Hmo1 antibodies [S2] as loading controls for the soluble fraction and chromatin pellet, respectively.
(TIF)

Figure S6 Depletion of Scc3 and Pds5 does not affect cohesin association with chromatin. (A) Yeast strains 1759 (*SMC3-MYC18*), 1769 (*SMC3-MYC18, Δwpl1*), 1776 (*SMC3-MYC18, SCC3-HA6*-degron), 1779 (*SMC3-MYC18*, *PDS5-HA6*-degron), 2197 (*SMC3-MYC18, PDS5-HA6*), 2227 (*SMC3-MYC18, SCC3-HA6*) were staged in G1 with *α*-factor and released into media with nocodazole. Chromosomal spreads were prepared at indicated time points as in Figure 2. At every time point fluorescence of 50 nuclei was determined. Error bars represent standard deviation. Cellular DNA content was analyzed by FACS (B).
(TIF)

Figure S7 Scc3 and Pds5 associate with chromosomes in Scc1-dependent manner. (A) Strains 1835 (*GAL-SCC1-Myc18*, *SCC3-HA6*), 1813 (*SCC1-Myc18*, *SCC3-HA6*), 1839 (*GAL-SCC1-Myc18*, *PDS5-HA6*), and 1815 (*SCC1-Myc18*, *PDS5-HA6*) were grown in media with galactose and arrested with *α*-factor for 2 hours. Media was then changed to YEP glucose and cells were incubated in the presence of *α*-factor for additional 60 minutes before release in YEP glucose with nocodazole. Chromosomal spreads were prepared at indicated times as in Figure 2. Samples from the same experiment were processed for Western blot shown in (B) and FACS analysis of cellular DNA content shown in (C).
(TIF)

Figure S8 ChIP-qPCR assay of Scc1. (A) Strains 10589 (wild type), 1625 (*SCC3*-degron), 1818 (*PDS5*-degron), 1906 (*Δwpl1*) with endogenous *SCC1* tagged with Myc18, and untagged strain (1021) were arrested with nocodazole. Chromatin immunoprecipitation was performed with anti-Myc and anti-histone H3 antibodies. ChIP DNA was quantified by quantitative PCR using 3 pairs of primers amplifying centromere adjacent region of chromosome VI, cohesin-high site on the arm of chromosome VI (172 kb), and cohesin-low site on the arm of chromosome V (141 kb). (B) The immunoprecipitation ratios of Scc1 were normalized between the strains using the control IP ratios of H3 and divided by the resultant IP ratio of wild type. (C) Schematic of the analyzed chromosomal regions.
(TIF)

Figure S9 Scc3, Pds5, and Wpl1 depletion does not affect the genome-wide distribution of cohesin. (A) Scc1 distribution on chromosome VII is shown. A window of 5.000 bps (i.e., 2.500 bps in each direction) was used for smoothing. The data sets for the wild type and one of the mutant strains are plotted on the same graph to facilitate comparison. (B) Scc1 distribution at the tDNA loci of chromosomes VII. Position of the tDNA genes is marked with red lines. A window of 500 bp with a 50 bp step was used.
(TIF)

Figure S10 Depletion of Scc3, Pds5, and Wpl1 does not affect Smc3 turnover rate on chromosomes. A FRAP experiment was performed on mitotic cells of 2003 (wild type), 2040 (*SCC3*-degron), 2004 (*PDS5*-degron), and 2034 (*Δwpl1*) strains with endogenous *SMC3* tagged with GFP. No recovery of the bleached pericentric cohesin was observed during experiment. The mean and standard deviation were calculated from independent experiments (numbers of observed cells for each strain are indicted on the graphs).
(TIF)

Figure S11 Specificity of the rabbit polyclonal anti-acetyl Smc3 antibody. The antibody was raised against a peptide CRTVGLK(Ac)K(Ac)DDYQL and affinity purified (Eurogentec). Strains 1021 (wild type), 1759 (*SMC3-MYC18*), 1752 (*Δwpl1, Δeco1*) and 1578 (*smc3* (K113N)) were grown until early log phase in YEPD. TCA protein extracts were analyzed by Western blot. The * indicates a non-specific band.
(TIF)

Figure S12 Chromosomal loss and X-ray sensitivity of strains depleted of Scc3 and Pds5. (A) Chromosomal loss in *SCC3*-degron strain. Cell suspensions containing 12×10^6^ cells from 1480 (*SCC3-HA6::HIS3*) and 1326 (*SCC3*-degron::*NAT*) *MAT α* strains were mixed with an equivalent number of cells from *MAT α* or *MAT a* tester strains (*his1*, otherwise prototrophic) on Millipore nitrocellulose filters. Filters were placed on YEPD plates and incubated for 8 hours at 25°C. Cells were washed off the surface of the filter, diluted and plated on minimal media to select for diploids and selective media to select for diploids and one of the parents. Selective media was media without histidine to select for *SCC3-HA6::HIS3* or YEPD with nourseothricin to select for *SCC3*-degron::*NAT*. The mating titer was calculated from the ratio between the numbers of colonies on minimal versus selective plates and dilution factor. The error bars represent standard deviation calculated from the results of two independent experiments. (B) Depletion of Scc3 and Pds5 results in X-ray hypersensitivity. Exponentially growing strains 1021 (wild type), 1366 (Δ*wpl1*), 1323 (*SCC3*-degron), and 1675 (*PDS5*-degron) were plated on YEPD and exposed to X-ray irradiation for 40, 80, 120 and 160 minutes. Emerging colonies were counted after two days incubation and % survival was calculated.
(TIF)

Figure S13 FACS analysis of cellular DNA content from the experiment in Figure 3C and Figure 5.
(TIF)

Table S1 List of yeast strains.
(DOC)

Table S2 Summary of ChIP-seq reads alignment against the Saccharomyces cerevisiae genome.
(DOC)
