# Supplementary figures and images for: Cohesin Rings Devoid of Scc3 and Pds5 Maintain Their Stable Association with the DNA
Source: PLoS Genet. 2012 Aug 9;8(8):e1002856. doi: 10.1371/journal.pgen.1002856 (PMC3415457; doi:10.1371/journal.pgen.1002856)

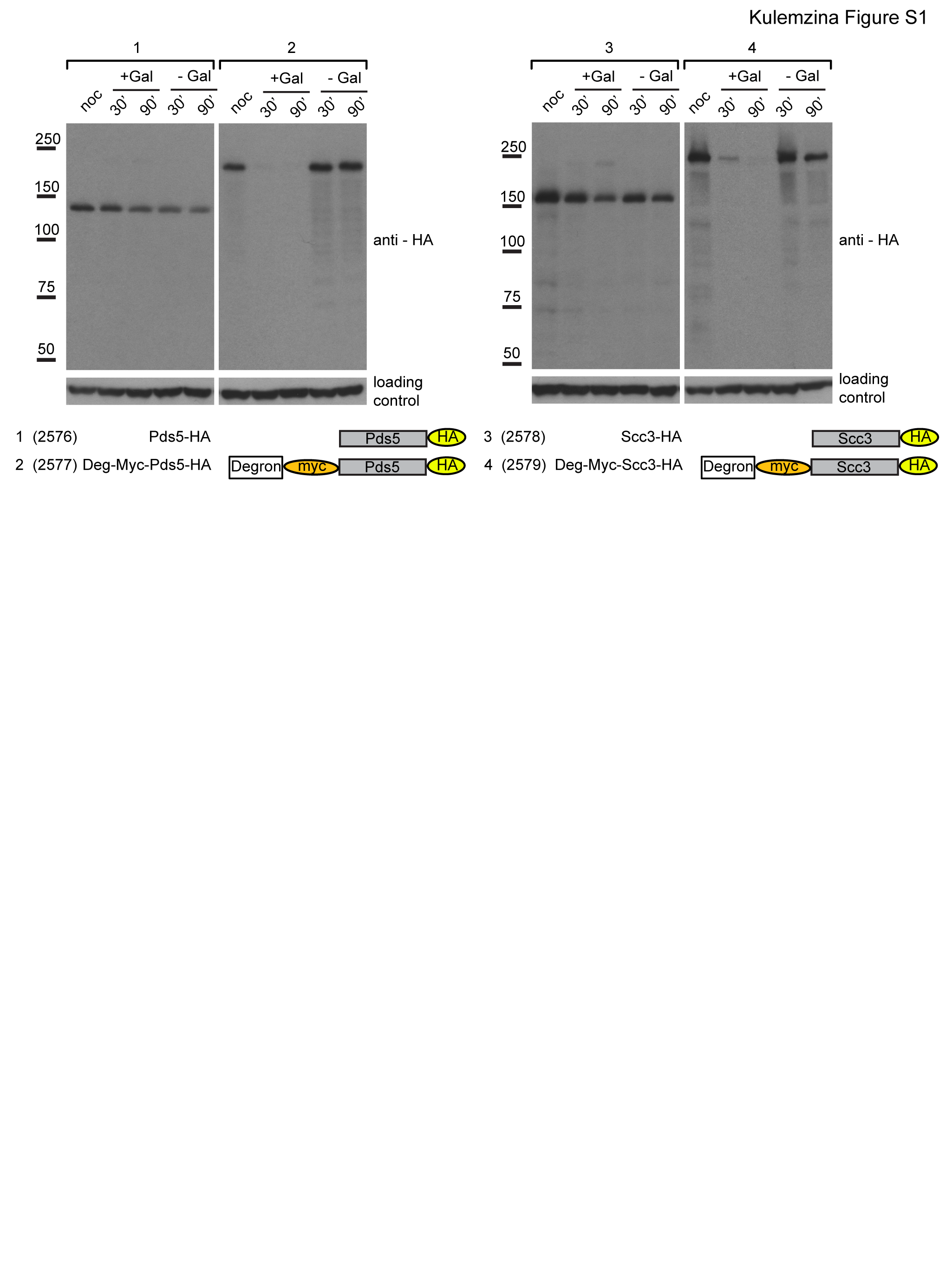

Supplement: Figure S1 — An induction of the “conventional” N-terminal ts degron results in a complete degradation of Pds5 and Scc3. To test if any stable fragments were remaining after the degron induction PDS5 and SCC3 were tagged at the C-termini with HA6. Degrons were induced in nocodazole-arrested yeast as in Figure 2A–C. Strain numbers are indicated in brackets in the figure. Western blots probed with anti-HA (16B12) antibody and anti-Cdc28 (sc-28550, Santa Cruz) as a loading control are shown. No stable fragments could be detected. (TIF) [file pgen.1002856.s001.tif]

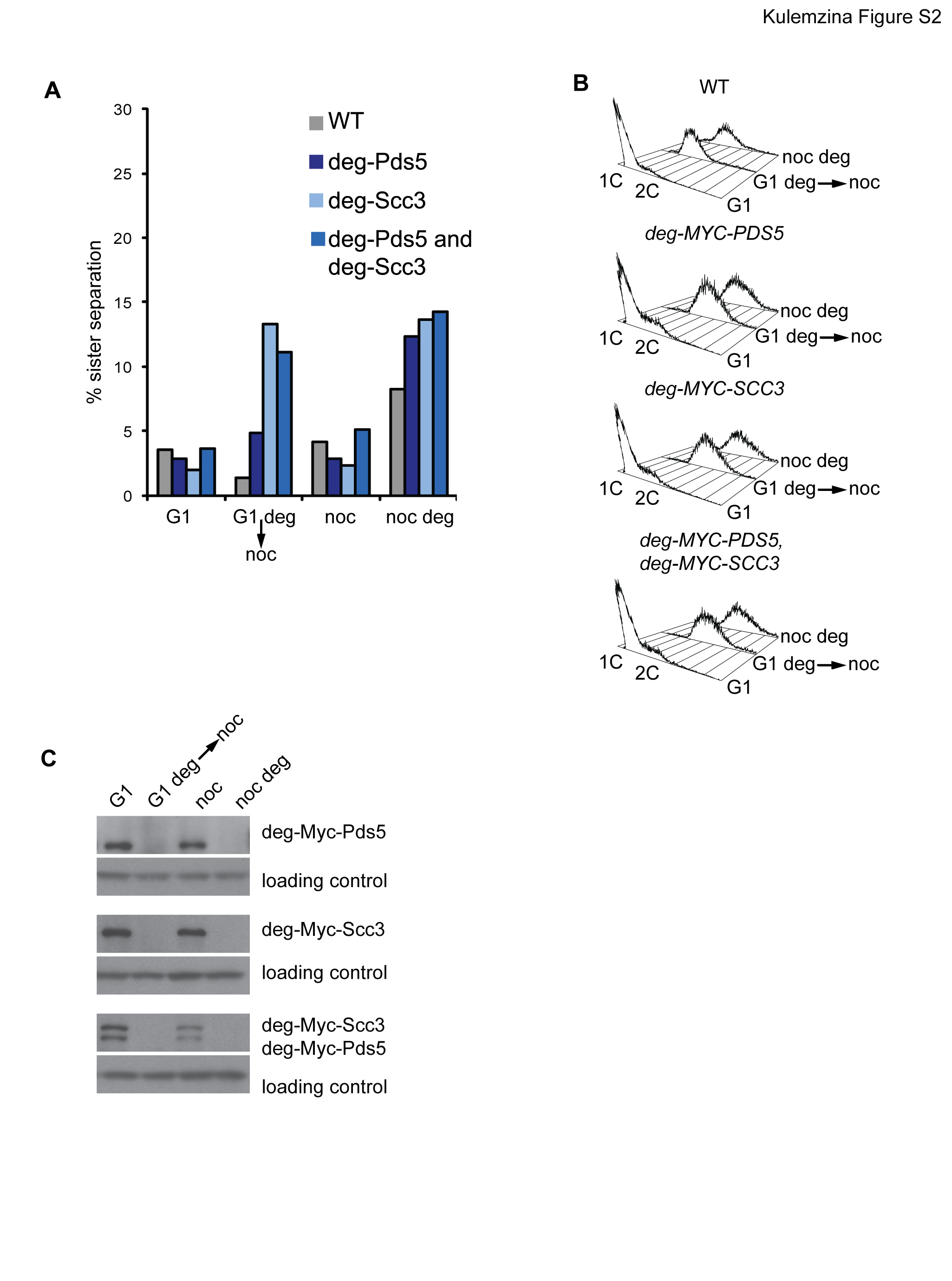

Supplement: Figure S2 — Sister chromatid cohesion defect in yeast depleted of Scc3 and Pds5 using a “conventional” N-terminal ts degron. (A) Strains 2418 (wild type), 2419 (degron-PDS5), 2420 (degron-SCC3), and 2449 (degron-PDS5 and degron-SCC3) have an array of Lac operators integrated into URA3 locus 35 kb from the centromere on chromosome V and express LacI-GFP. To induce the degron in G1, strains were staged with α-factor in YEP raffinose. Cells were resuspended in YEP galactose containing α-factor and incubated for 45 minutes at 30°C to induce the expression of Ubr1. Cells were then shifted to 37°C in YEP galactose containing doxycycline and α-factor and incubated for additional 90 minutes to destroy Pds5 and/or Scc3. Cells were released from α-factor arrest into YEP galactose containing nocodazole and doxycycline at 25°C for 3 hours. To induce degron in G2/M, strains were synchronized with α-factor and released into nocodazole containing YEP raffinose medium for 2 hours. Cells were resuspended in YEP galactose containing nocodazole and incubated for 45 minutes at 30°C to induce the expression of Ubr1. Cells were then shifted to 37°C in YEP galactose containing doxycycline and nocodazole and incubated for 90 minutes to destroy Pds5 and/or Scc3. Cells were then chased in YEP galactose containing nocodazole and doxycycline at 25°C for 3 hours. This last incubation step was found necessary since the dots signal was weakened under the conditions of degron induction. Separation of sister chromatids was scored as one (non-separated) versus two (separated) GFP dots in 300 cells. (B) FACS analysis of cellular DNA content. (C) Western blot demonstrating the depletion of Pds5 and Scc3. TCA protein extracts were prepared at indicated time points. Blots were probed with anti-Myc antibody (71D10) and anti-Cdc28 (sc-28550, Santa Cruz) for loading control. (TIF) [file pgen.1002856.s002.tif]

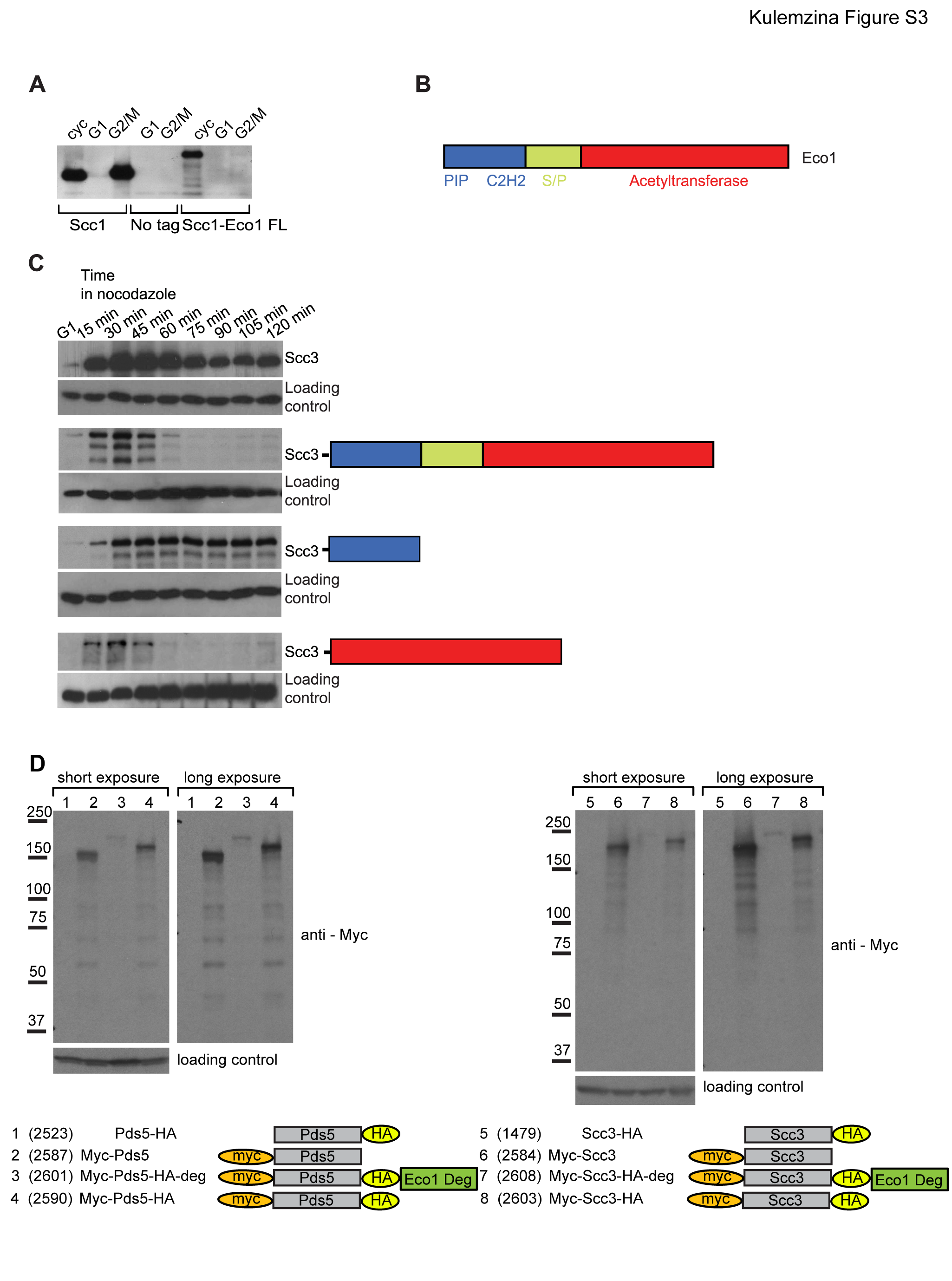

Supplement: Figure S3 — Eco1 contains degron sequences. (A) 1188 (GAL-SCC1), 1176 (GAL-SCC1 SCC1-HA6) and 1177 (GAL-SCC1 SCC1-HA6-ECO1) strains were synchronized in G1 with α-factor and then released into media with glucose and nocodazole for 90 minutes. Western blot was probed with anti-HA antibody (12CA5). (B) Schematic representation of Eco1 domains. N-terminal region with the PCNA-interacting PIP box and C2H2 Zinc finger, middle region rich in serines and prolines and C-terminal acetyltransferase domain are indicated. (C) GAL-SCC3 strains carrying transgenes SCC3-HA6 (1257), SCC3-HA6-ECO1 (1258), SCC3-HA6-ECO1(aa1–63) (1259) and SCC3-HA6-ECO1(aa111–281) (1260) were staged in G1 with α-factor and then released into galactose-containing media with nocodazole. Western blot was probed with anti-HA antibody (12CA5) to detect Scc3 or Scc3-Eco1 fusions. Loading control was anti-Cdc28 sc-28550 (Santa Cruz). (D) No stable fragments of Pds5 and Scc3 can be detected in the degron strains. PDS5 and SCC3 were tagged at the N-termini with Myc9. Strains were arrested in G2/M with nocodazole for 3 hours and TCA protein extracts were prepared. Strain numbers are indicated in brackets in the figure. Western blots probed with anti-Myc (71D10) antibody and anti-Cdc28 (sc-28550, Santa Cruz) as a loading control are shown. (TIF) [file pgen.1002856.s003.tif]

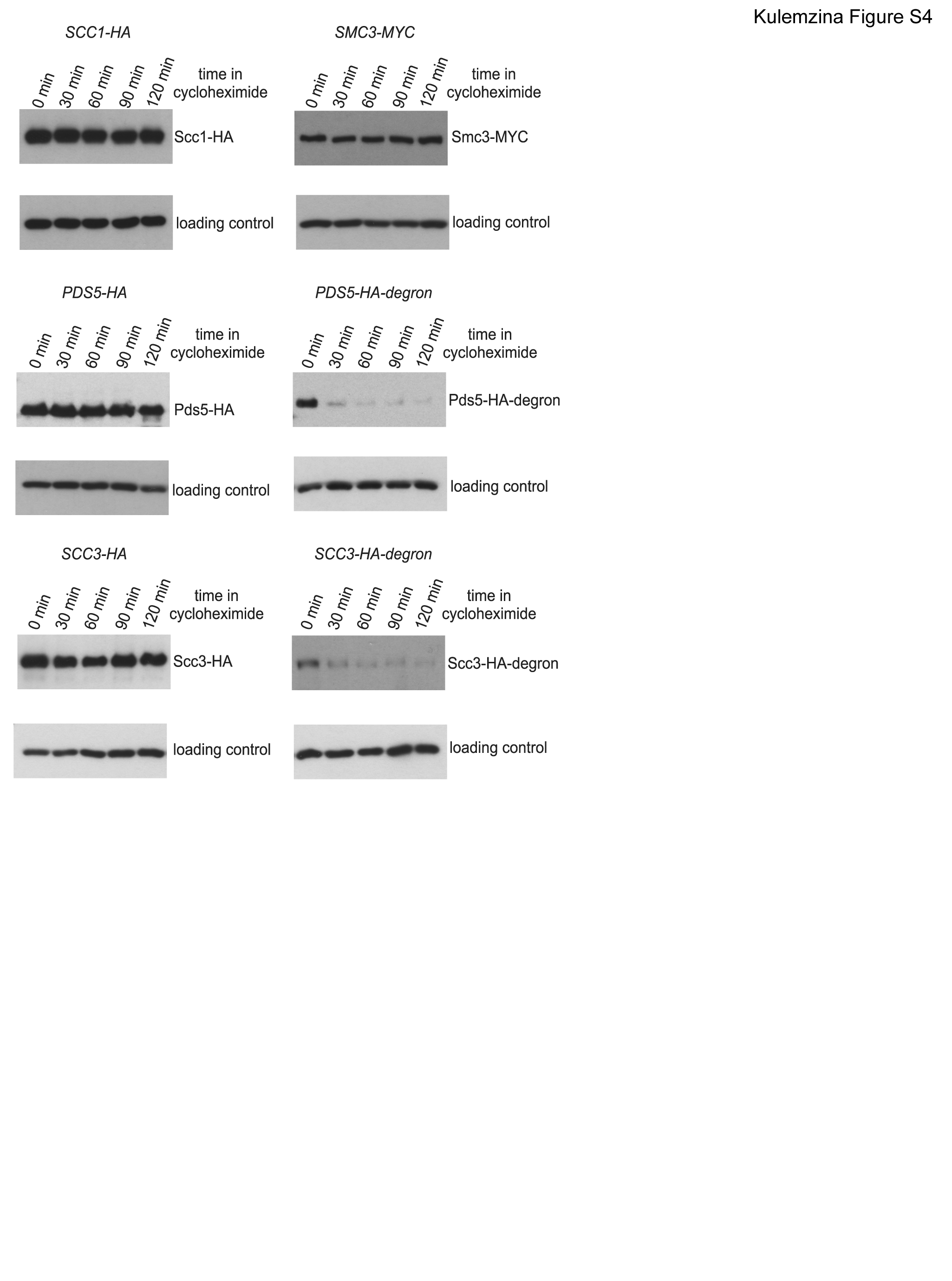

Supplement: Figure S4 — Protein stability assay in nocodazole arrested cells. Strains 1323 (SCC3-HA6-degron), 1479 (SCC3-HA6), 1675 (PDS5-HA6-degron), 1677 (PDS5-HA6), 1759 (SMC3-MYC18), and 10589 (SCC1-HA6) were arrested with nocodazole for 2 hours. Cycloheximide was added at a final concentration of 0.1 mg/ml, samples were taken at the indicated time points and TCA protein extracts prepared. Western blot was probed with anti-HA (16B12) or anti-MYC (71D10) and anti-Cdc28 (sc-28550, Santa Cruz) antibodies as a loading control. (TIF) [file pgen.1002856.s004.tif]

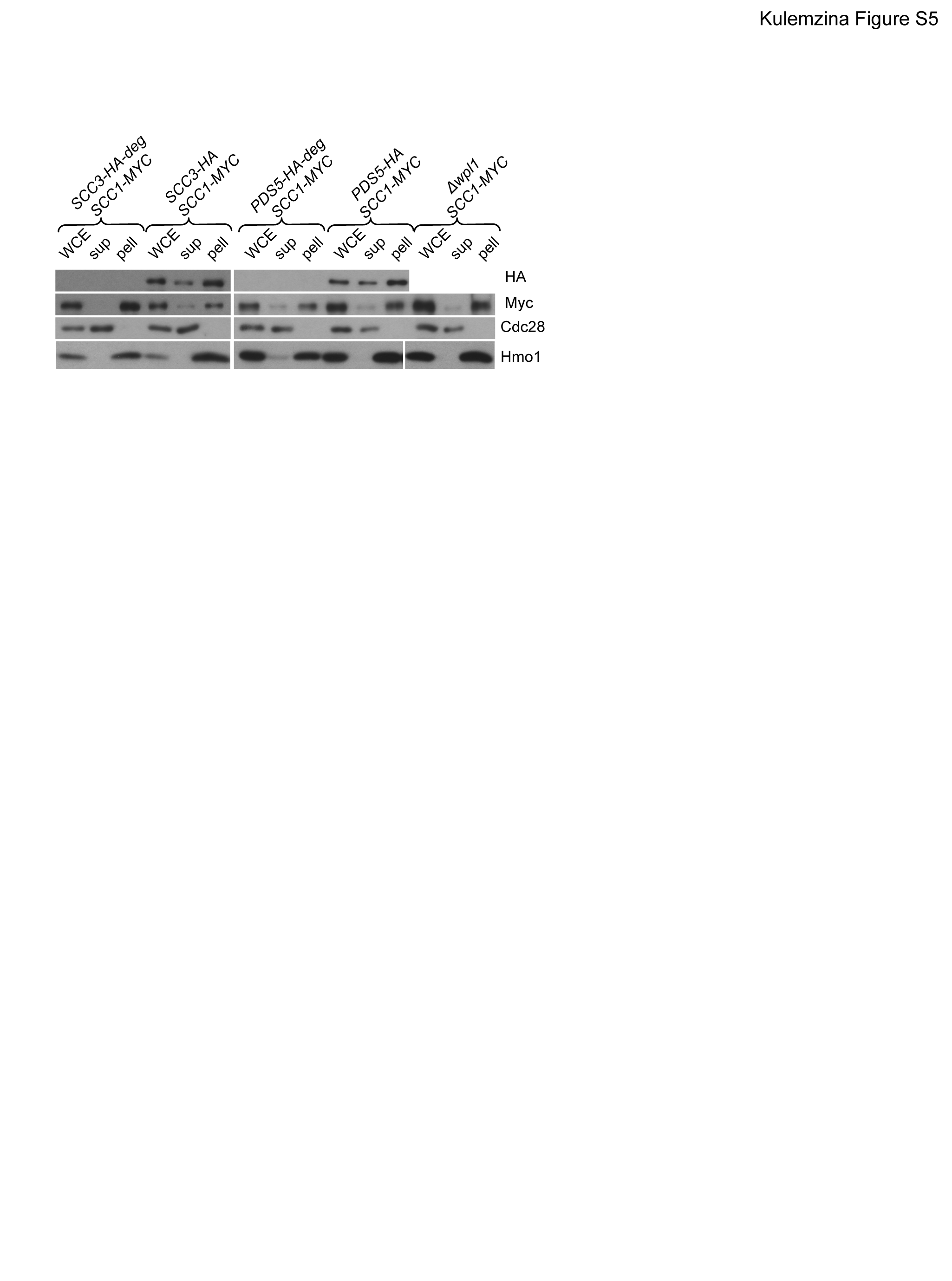

Supplement: Figure S5 — Chromatin-bound fraction of cohesin is not affected by the depletion of Scc3 and Pds5. Strains 1813 (SCC1-Myc18, SCC3-HA6), 1625 (SCC1-Myc18, SCC3-HA6-degron), 2525 (SCC1-Myc18, PDS5-HA6), 1818 (SCC1-Myc18, PDS5-HA6-degron) and 1906 (SCC1-Myc18, Δwpl1) were arrested in G2/M with nocodazole. Whole cell extracts (WCE) were fractionated into soluble supernatant (sup) and chromatin pellet (pell). Equivalent amounts of protein samples were separated on SDS-PAGE. Western blot was probed with anti-HA (16B12), anti-Myc (71D10), as well as with anti-Cdc28 (sc-28550, Santa Cruz) and anti-Hmo1 antibodies [S2] as loading controls for the soluble fraction and chromatin pellet, respectively. (TIF) [file pgen.1002856.s005.tif]

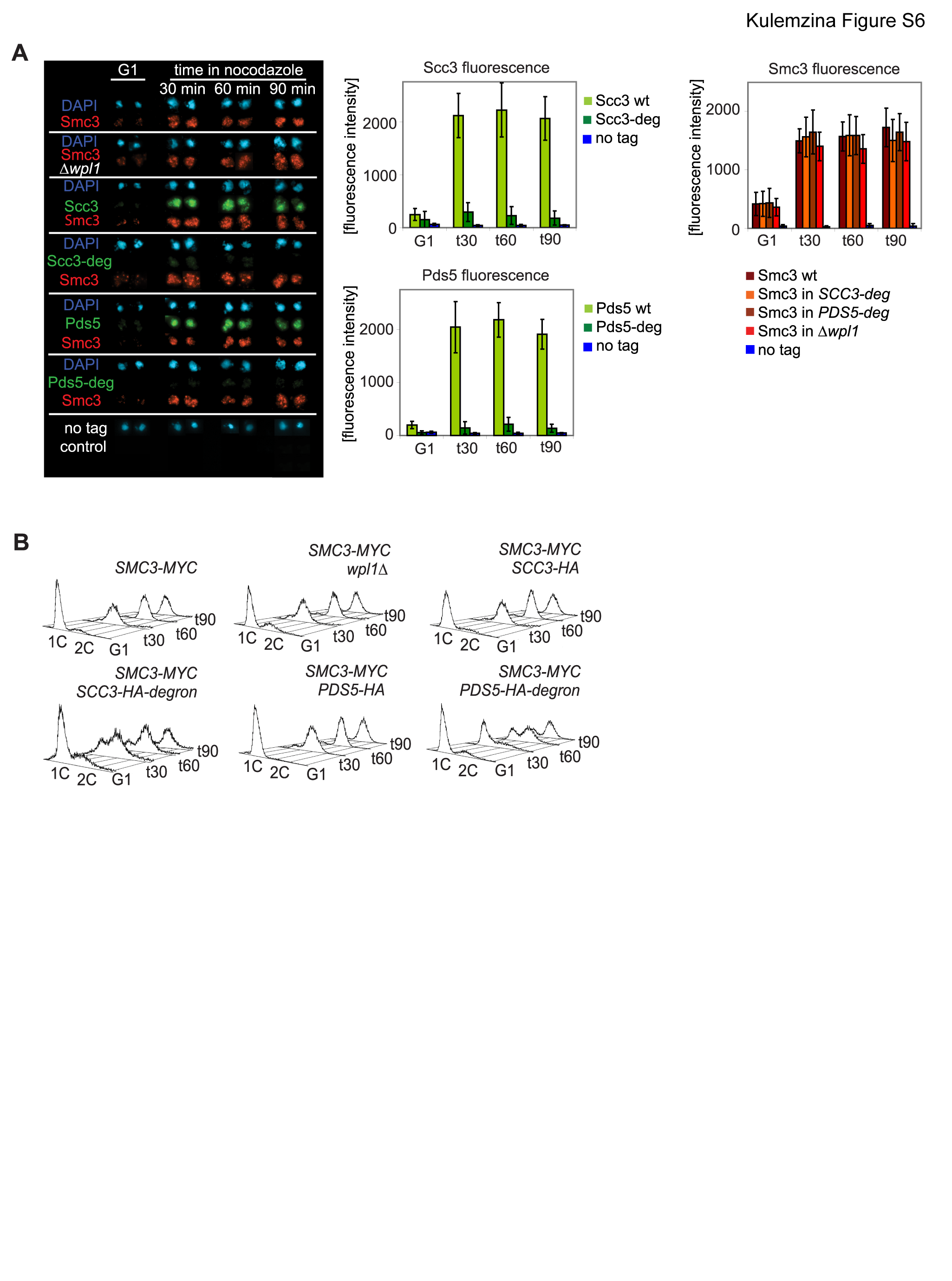

Supplement: Figure S6 — Depletion of Scc3 and Pds5 does not affect cohesin association with chromatin. (A) Yeast strains 1759 (SMC3-MYC18), 1769 (SMC3-MYC18, Δwpl1), 1776 (SMC3-MYC18, SCC3-HA6-degron), 1779 (SMC3-MYC18, PDS5-HA6-degron), 2197 (SMC3-MYC18, PDS5-HA6), 2227 (SMC3-MYC18, SCC3-HA6) were staged in G1 with α-factor and released into media with nocodazole. Chromosomal spreads were prepared at indicated time points as in Figure 2. At every time point fluorescence of 50 nuclei was determined. Error bars represent standard deviation. Cellular DNA content was analyzed by FACS (B). (TIF) [file pgen.1002856.s006.tif]

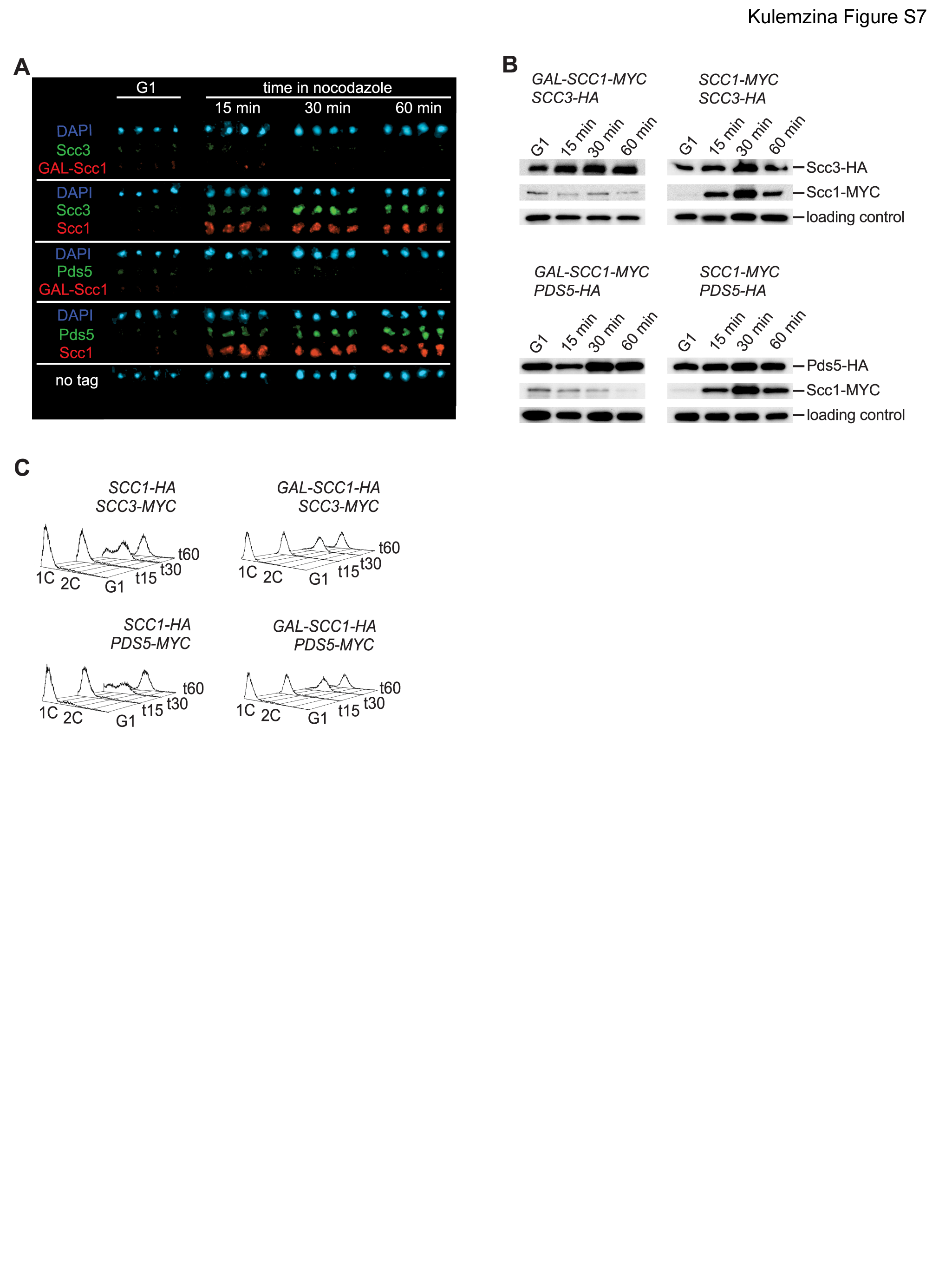

Supplement: Figure S7 — Scc3 and Pds5 associate with chromosomes in Scc1-dependent manner. (A) Strains 1835 (GAL-SCC1-Myc18, SCC3-HA6), 1813 (SCC1-Myc18, SCC3-HA6), 1839 (GAL-SCC1-Myc18, PDS5-HA6), and 1815 (SCC1-Myc18, PDS5-HA6) were grown in media with galactose and arrested with α-factor for 2 hours. Media was then changed to YEP glucose and cells were incubated in the presence of α-factor for additional 60 minutes before release in YEP glucose with nocodazole. Chromosomal spreads were prepared at indicated times as in Figure 2. Samples from the same experiment were processed for Western blot shown in (B) and FACS analysis of cellular DNA content shown in (C). (TIF) [file pgen.1002856.s007.tif]

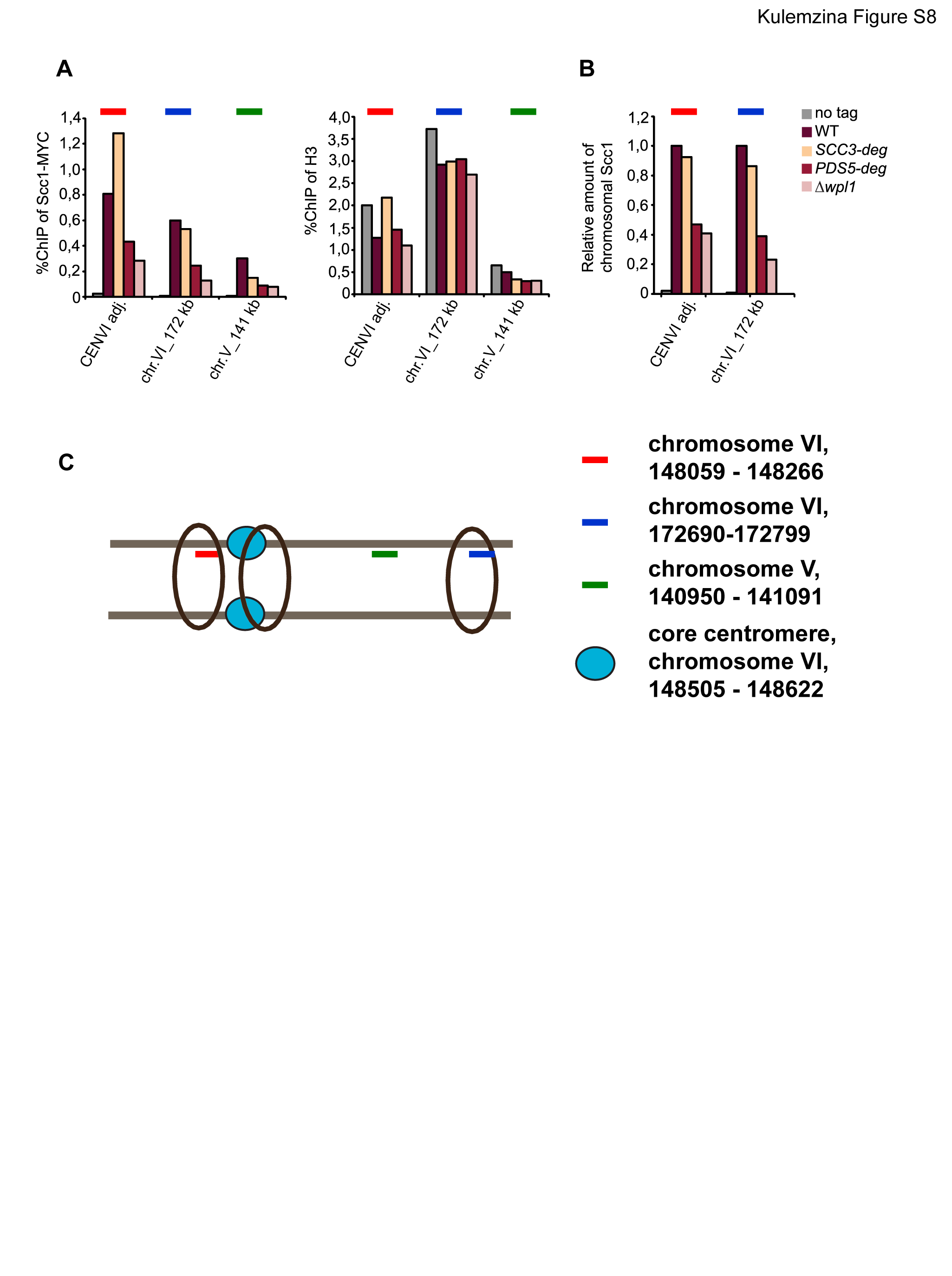

Supplement: Figure S8 — ChIP-qPCR assay of Scc1. (A) Strains 10589 (wild type), 1625 (SCC3-degron), 1818 (PDS5-degron), 1906 (Δwpl1) with endogenous SCC1 tagged with Myc18, and untagged strain (1021) were arrested with nocodazole. Chromatin immunoprecipitation was performed with anti-Myc and anti-histone H3 antibodies. ChIP DNA was quantified by quantitative PCR using 3 pairs of primers amplifying centromere adjacent region of chromosome VI, cohesin-high site on the arm of chromosome VI (172 kb), and cohesin-low site on the arm of chromosome V (141 kb). (B) The immunoprecipitation ratios of Scc1 were normalized between the strains using the control IP ratios of H3 and divided by the resultant IP ratio of wild type. (C) Schematic of the analyzed chromosomal regions. (TIF) [file pgen.1002856.s008.tif]

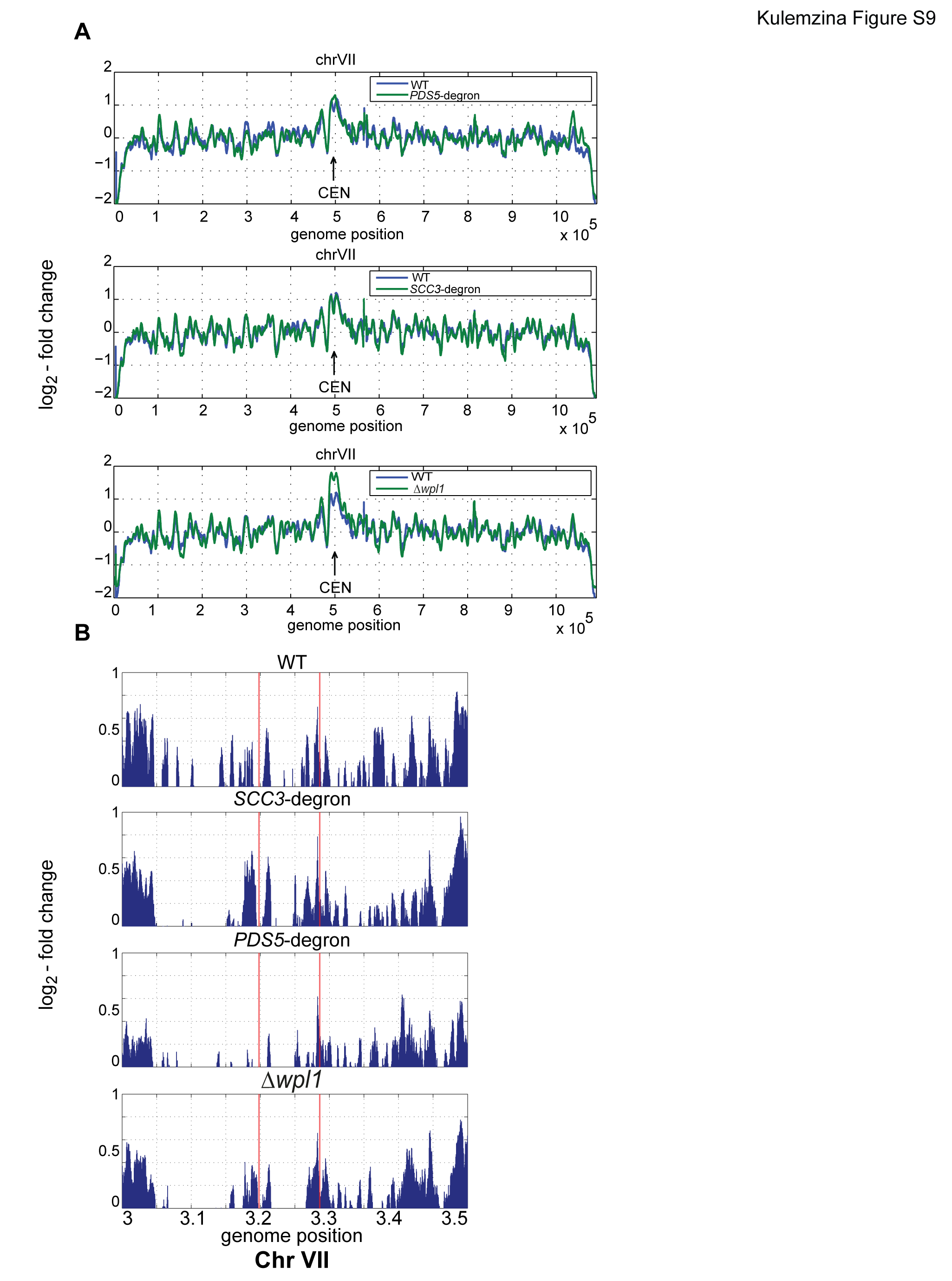

Supplement: Figure S9 — Scc3, Pds5, and Wpl1 depletion does not affect the genome-wide distribution of cohesin. (A) Scc1 distribution on chromosome VII is shown. A window of 5.000 bps (i.e., 2.500 bps in each direction) was used for smoothing. The data sets for the wild type and one of the mutant strains are plotted on the same graph to facilitate comparison. (B) Scc1 distribution at the tDNA loci of chromosomes VII. Position of the tDNA genes is marked with red lines. A window of 500 bp with a 50 bp step was used. (TIF) [file pgen.1002856.s009.tif]

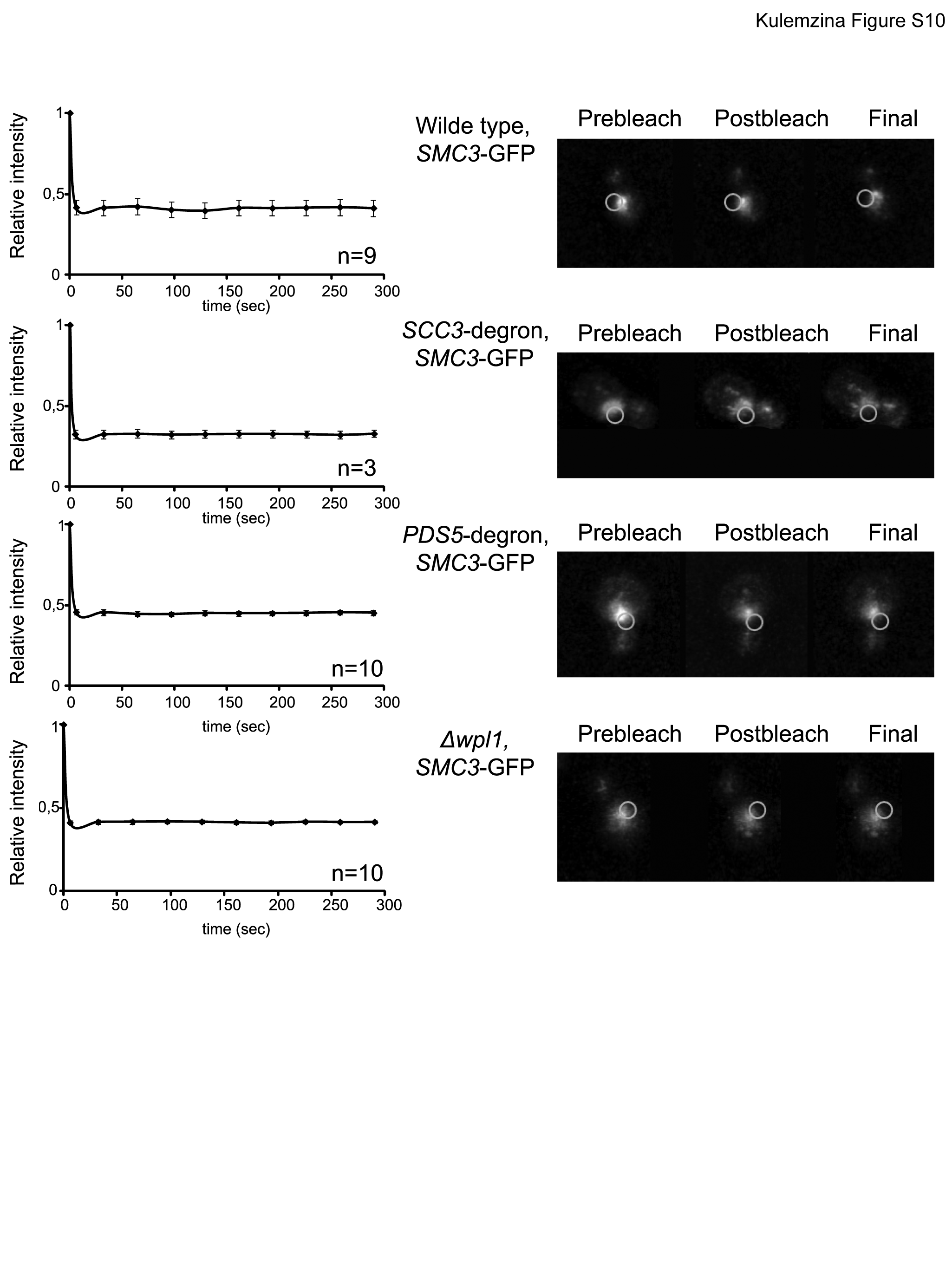

Supplement: Figure S10 — Depletion of Scc3, Pds5, and Wpl1 does not affect Smc3 turnover rate on chromosomes. A FRAP experiment was performed on mitotic cells of 2003 (wild type), 2040 (SCC3-degron), 2004 (PDS5-degron), and 2034 (Δwpl1) strains with endogenous SMC3 tagged with GFP. No recovery of the bleached pericentric cohesin was observed during experiment. The mean and standard deviation were calculated from independent experiments (numbers of observed cells for each strain are indicted on the graphs). (TIF) [file pgen.1002856.s010.tif]

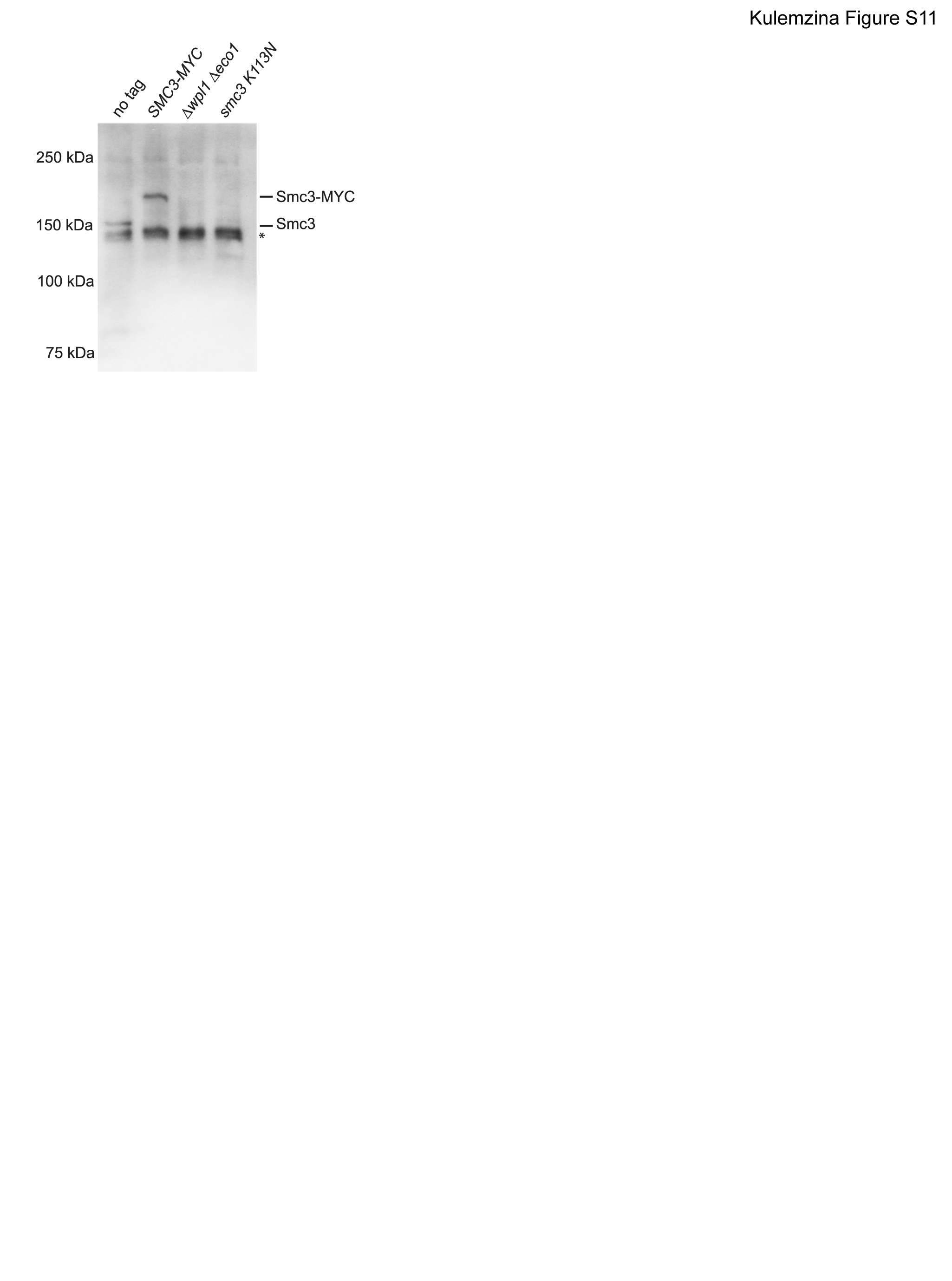

Supplement: Figure S11 — Specificity of the rabbit polyclonal anti-acetyl Smc3 antibody. The antibody was raised against a peptide CRTVGLK(Ac)K(Ac)DDYQL and affinity purified (Eurogentec). Strains 1021 (wild type), 1759 (SMC3-MYC18), 1752 (Δwpl1, Δeco1) and 1578 (smc3 (K113N)) were grown until early log phase in YEPD. TCA protein extracts were analyzed by Western blot. The * indicates a non-specific band. (TIF) [file pgen.1002856.s011.tif]

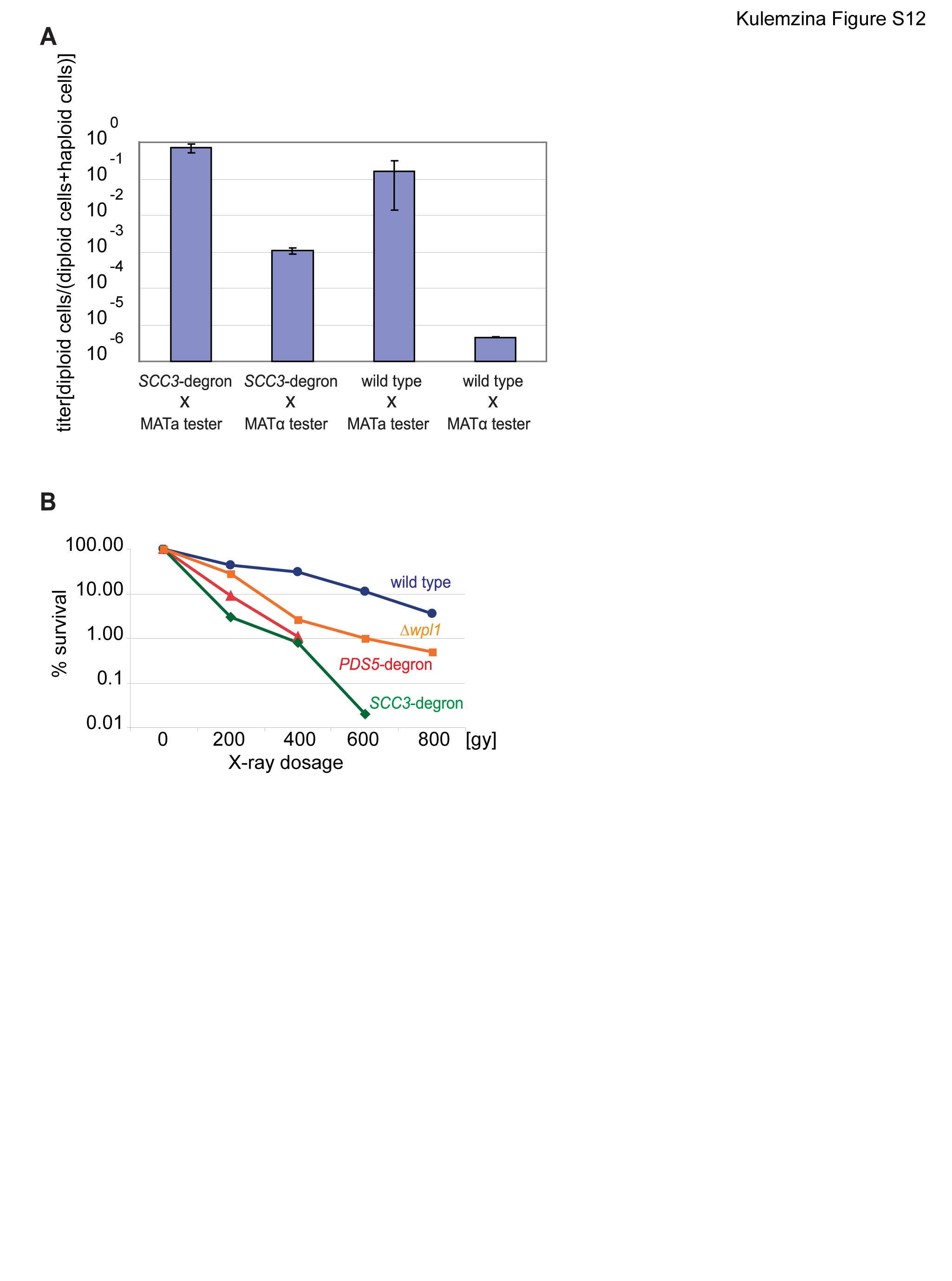

Supplement: Figure S12 — Chromosomal loss and X-ray sensitivity of strains depleted of Scc3 and Pds5. (A) Chromosomal loss in SCC3-degron strain. Cell suspensions containing 12×106 cells from 1480 (SCC3-HA6::HIS3) and 1326 (SCC3-degron::NAT) MAT α strains were mixed with an equivalent number of cells from MAT α or MAT a tester strains (his1, otherwise prototrophic) on Millipore nitrocellulose filters. Filters were placed on YEPD plates and incubated for 8 hours at 25°C. Cells were washed off the surface of the filter, diluted and plated on minimal media to select for diploids and selective media to select for diploids and one of the parents. Selective media was media without histidine to select for SCC3-HA6::HIS3 or YEPD with nourseothricin to select for SCC3-degron::NAT. The mating titer was calculated from the ratio between the numbers of colonies on minimal versus selective plates and dilution factor. The error bars represent standard deviation calculated from the results of two independent experiments. (B) Depletion of Scc3 and Pds5 results in X-ray hypersensitivity. Exponentially growing strains 1021 (wild type), 1366 (Δwpl1), 1323 (SCC3-degron), and 1675 (PDS5-degron) were plated on YEPD and exposed to X-ray irradiation for 40, 80, 120 and 160 minutes. Emerging colonies were counted after two days incubation and % survival was calculated. (TIF) [file pgen.1002856.s012.tif]

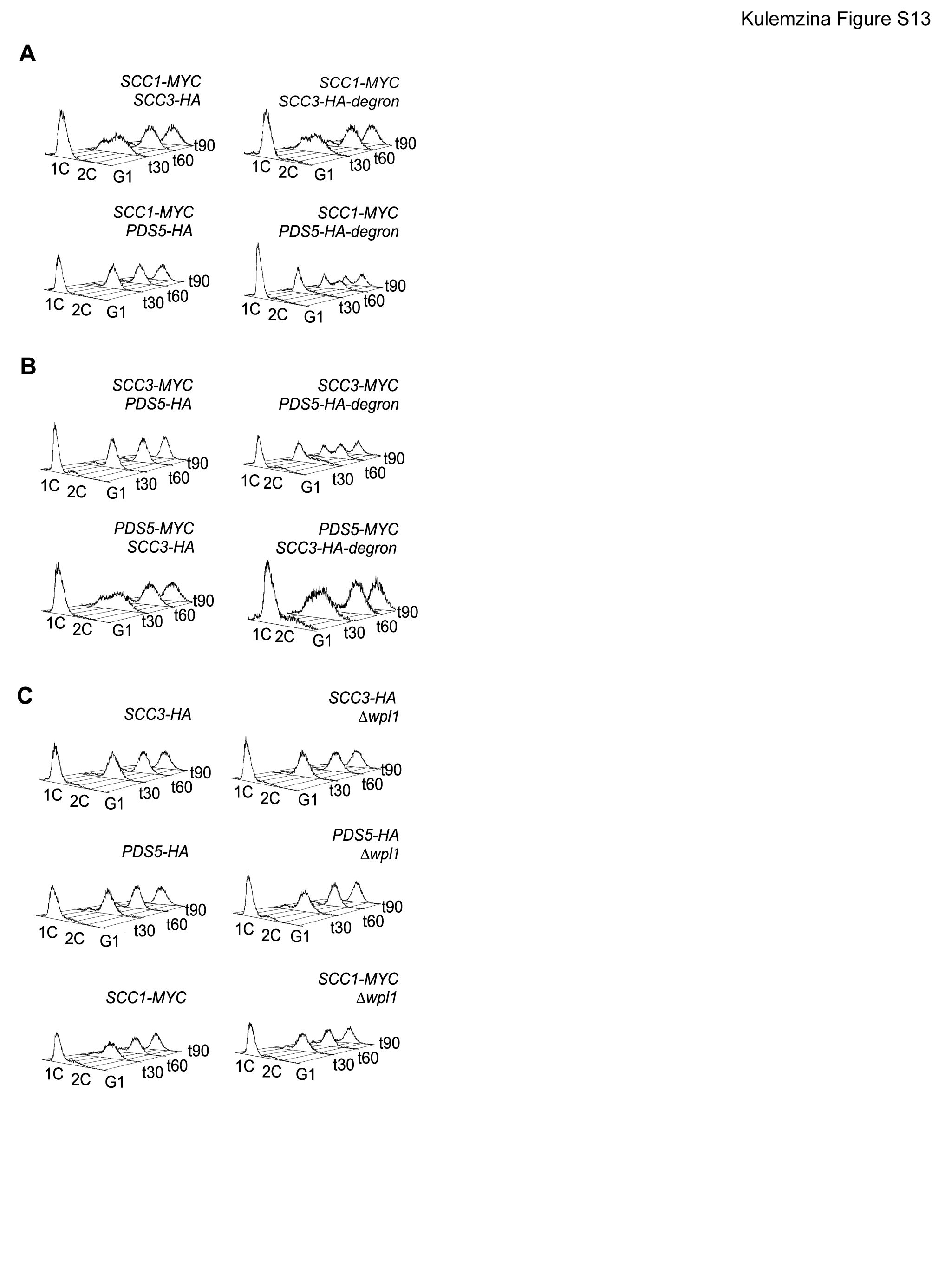

Supplement: Figure S13 — FACS analysis of cellular DNA content from the experiment in Figure 3C and Figure 5. (TIF) [file pgen.1002856.s013.tif]
