# Supplementary material for: Cohesin Rings Devoid of Scc3 and Pds5 Maintain Their Stable Association with the DNA
Source: PLoS Genet. 2012 Aug 9;8(8):e1002856. doi: 10.1371/journal.pgen.1002856 (PMC3415457; doi:10.1371/journal.pgen.1002856)
Supplement: Table S1 — List of yeast strains. (DOC) [file pgen.1002856.s014.doc]

**Table S1. List of yeast strains**

| Strain | Genotype*^a^* |
| --- | --- |
| 1021 | Wild type |
| 1188 | *scc1::GAL1-SCC1::TRP1* |
| 1176 | *scc1::GAL1-SCC1::TRP1 ura3::SCC1-HA6::URA3* |
| 1177 | *scc1::GAL1-SCC1::TRP ura3::SCC1-HA6-ECO1::URA3* |
| 1222 | *ECO1-HA-TAP::TRP1 cdc20::MET-CDC20::LEU2* |
| 1257 | *scc3:: GAL1-SCC3:TRP1* *ura3::SCC3-HA6::URA3* |
| 1258 | *scc3:: GAL1-SCC3:TRP1* *ura3::SCC3-HA6-ECO1::URA3* |
| 1259 | *scc3:: GAL1-SCC3:TRP1*  *ura3::SCC3-HA6-ECO1(aa1-63)::URA3* |
| 1260 | *scc3:: GAL1-SCC3:TRP1*  *ura3::SCC3-HA6-ECO1(aa111-281)::URA3* |
| 1264 | *scc3:: GAL1-SCC3:TRP1*  *ura3::SCC3-HA6-ECO1(aa63-109)::URA3* |
| 1323 | *SCC3-HA6-ECO1(aa63-109)::NAT* |
| 1326 | *MATα SCC3-HA6-ECO1(aa63-109)::NAT* |
| 1366 | *wpl1::HPH* |
| 1417 | *TetO200::URA3 TetR-GFP::LEU2* |
| 1479 | *SCC3-HA6::HIS3* |
| 1480 | *MATα SCC3-HA6::HIS3* |
| 1578  (courtesy of Frank Uhlmann) | *SMC3(K113N)::TRP1 ura3::3XURA3 tetO112*  *his3::HIS tetR-GFP* |
| 1621 | *TetR-GFP::LEU2 TetO200::URA3*  *SCC3-HA6-ECO1(aa63-109)::NAT,* |
| 1625 | *SCC3-HA6-ECO1(aa63-109)::NAT SCC1-Myc18::HIS3* |
| 1675 | *PDS5-HA6-ECOI(aa63-109) :: NAT* |
| 1677 | *PDS5-HA6::TRP1* |
| 1678 | *TetO200::URA3, TetR-GFP::LEU2*  *PDS5-HA6-ECOI(aa63-109)::NAT* |
| 1734 | *PDS5-Myc18::TRP1 SCC3-HA6::HIS3* |
| 1744 | *SCC3-HA6-ECO1(aa63-109)::NAT PDS5-Myc18::TRP1* |
| 1752 | *wpl1::HPH eco1::LEU2* |
| 1759 | *SMC3-Myc18::HIS3* |
| 1769 | *SMC3-Myc18::HIS3 wpl1::HPH* |
| 1771 | *PDS5-HA6::TRP1 SCC3-Myc18::HIS3* |
| 1776 | *SMC3-Myc18::HIS3 SCC3-HA6-ECO1(aa63-109)::NAT* |
| 1779 | *SMC3-Myc18::HIS3 PDS5-HA6-ECO1(aa63-109)::NAT* |
| 1796 | *PDS5-HA6-ECO1(aa63-109)::NAT SCC3-Myc18::HIS3* |
| 1813 | *SCC3-HA6::HIS3 SCC1-Myc18::HIS3* |
| 1815 | *SCC1-Myc18::HIS3 PDS5-HA6::TRP1* |
| 1818 | *PDS5-HA6-ECO1(aa63-109)::NAT SCC1-Myc18::HIS3* |
| 1822  (JF152 [S1]) | *TetR-GFP::LEU2 CENIV::TetO448::URA3*  *cdc20::MET-CDC20::URA SPC42-Tomato::NAT* |
| 1829 | *SCC1-HA-TAP::TRP1 PDS5-HA6-ECO1(aa63-109)::NAT*  *SCC3-Myc18::HIS3 TetR-GFP::LEU2 TetO200::URA3* |
| 1832 | *TetR-GFP::LEU2 CENIV::TetO448::URA3*  *cdc20::MET-CDC20::URA SPC42-Tomato::NAT*  *SCC3-HA6-ECO1(aa63-109)::NAT* |
| 1833 | *TetR-GFP::LEU2 CENIV::TetO448::URA3*  *cdc20::MET-CDC20::URA SPC42-Tomato::NAT*  *PDS5-HA6-ECO1(aa63-109)::NAT* |
| 1834 | *SCC1-HA-TAP::TRP1 SCC3-HA6-ECO1(aa63-109)::NAT PDS5-Myc18::TRP1* |
| 1835 | *scc1:: TRP1::GAL1-SCC1-Myc18:: HIS3*  *SCC3-HA6::HIS3* |
| 1839 | *scc1:: TRP1::GAL1-SCC1-Myc18:: HIS3*  *PDS5-HA6::TRP1* |
| 1864 | *wpl1::HPH SCC3-HA6::HIS3* |
| 1866 | *wpl1::HPH PDS5-HA6::TRP1* |
| 1880 | *SCC3-HA6::HIS3 WPL1-Myc18::HIS3* |
| 1882 | *PDS5-HA6::TRP1 WPL1-Myc18::HIS3* |
| 1904  (DCB 350.1, [S4]) | YEF473 background  *MATa trp1Δ63 leu2Δ1 ura3-52 his3Δ200 lys2-801*  *HTB2-GFP::KAN SPC29-RFP::HPH* |
| 1906 | *SCC1-Myc18::HIS3 wpl1::HPH* |
| 1956 | *SCC1-HA-TAP::TRP1 PDS5-Myc18::TRP1*  *SCC3-HA6::HIS3* |
| 1958 | *SCC1-HA-TAP::TRP1 SCC3-Myc18::HIS3*  *PDS5-HA6::TRP1* |
| 2003 | *MATa/α* diploid homozygous for  *SPC42-mCherry::NAT and SMC3-EGFP::KAN* |
| 2004 | *MATa/ α* diploid homozygous for  *SPC42-mCherry::NAT*, *SMC3-EGFP::KAN* and *PDS5-HA6-ECO1(aa63-109)::NAT* |
| 2012 | *SCC1-HA-TAP::TRP1 SCC3-HA6::HIS3*  *WPL1-Myc18::HIS3* |
| 2014 | *SCC1-HA-TAP::TRP1 PDS5-HA6::TRP1*  *WPL1-Myc18::HIS3* |
| 2016 | *SCC1-HA-TAP::TRP1 PDS5-HA6-ECO1(aa63-109)::NAT*  *WPL1-Myc18::HIS3* |
| 2018 | *SCC1-HA-TAP::TRP1 SCC3-HA6-ECO1(aa63-109)::NAT WPL1-Myc18::HIS3* |
| 2034 | *MATa/α* diploid homozygous for  *SPC42-mCherry::NAT*, *SMC3-EGFP::KAN* and *wpl1::HPH* |
| 2040 | *MATa/α* diploid *SPC42-mCherry::NAT/SPC42wt*  *SMC3-yEGFP::HYGRO/Smc3-EGFP::KAN*  homozygous for *SCC3-HA6-ECO1(aa66-109)::NAT* |
| 2190 | *wpl1::HPH TetO200::URA3 TetR-GFP::LEU2* |
| 2197 | *SMC3-Myc-18::HIS3 PDS5-HA6::TRP1* |
| 2227 | *SCC3-HA6::HIS3 SMC3-Myc18::HIS3* |
| 2249 | *SMC3-TAP::TRP1 PDS5-Myc18::TRP1 SCC3-HA6::HIS3* |
| 2251 | *SMC3-TAP::TRP1 PDS5-HA6::TRP1 SCC3-Myc18::HIS3* |
| 2253 | *SMC3-TAP::TRP1 PDS5-HA6::TRP1 WPL1-Myc18::HIS3* |
| 2261 | *SMC3-TAP::TRP1 SCC3-HA6::HIS3 WPL1-Myc18::HIS3* |
| 2264 | *SMC3-TAP::TRP1 SCC3-HA6-ECO1(aa63-109)::NAT*  *PDS5-Myc18::TRP1* |
| 2265 | *SMC3-TAP::TRP1 PDS5-HA6-ECO1(aa63-109)::NAT WPL1-Myc18::HIS3* |
| 2271 | *SMC3-TAP::TRP1 SCC3-HA6-ECO1(aa63-109)::NAT*  *WPL1-Myc18::HIS3* |
| 2281 | *MATa/α* diploid homozygous for  *SPC42-mCherry::NAT*  *SCC3-EGFP::KAN* |
| 2290 | *SMC3-TAP::TRP1 PDS5-HA6-ECO1(aa63-109)::NAT*  *SCC3-Myc18::HIS3* |
| 2353 | *MATa/α* diploid homozygous for  *SPC42-4mCherry::NAT* and *SCC1-EGFP::KAN* |
| 2389 | *MATa/α* diploid homozygous for  *PDS5-HA6-EcoI(aa63-109) :: NAT,*  *SPC42-mCherry::NAT*, and *SCC1-EGFP::KAN* |
| 2390 | *MATa/α* diploid homozygous for  *SCC3-HA6-ECO1(aa63-109)::NAT* and  *SCC1-EGFP::KAN* |
| 2391 | *MATa/α* diploid homozygous for  *wpl1::HPH*, *SPC42-mCherry::NAT*, and  *SCC1-EGFP::KAN* |
| 2395 | *MAT a*  *ubr1::GAL-HA-UBR1 (HIS3)*  *CMVp (tetR´-SSN6)::LEU2*  *trp1::tTA*  *SCC1-HA6::TRP1* |
| 2417 | *MATa/α* diploid homozygous for  *SPC42-mCherry::NAT* and  *PDS5-EGFP::KAN* |
| 2418 | *MATa*  *ubr1::GAL-HA-UBR1(HIS3)*  *CMVp(tetR'-SSN6)::LEU2*  *trp1::tTA*  *lacO repeat::URA*  *GFP-LacI::HIS3* |
| 2419 | *MAT a*  *ubr1::GAL-HA-UBR1 (HIS3)*  *CMVp (tetR´-SSN6)::LEU2*  *trp1::tTA*  *pds5::tet02-DHFR-Myc1-PDS5::KAN*  *lacO repeat::URA*  *GFP-LacI::HIS3* |
| 2420 | *MAT a*  *ubr1::GAL-HA-UBR1 (HIS3)*  *CMVp (tetR´-SSN6)::LEU2*  *trp1::tTA*  *scc3::tetO2-DHFR-Myc1-SCC3 ::KAN*  *lacO repeat::URA*  *GFP-LacI::HIS3* |
| 2449 | *MATa*  *ubr1::GAL-HA-UBR1 (HIS3)*  *CMVp (tetR´-SSN6)::LEU2*  *trp1::tTA*  *scc3::tetO2-DHFR-Myc1-SCC3 ::KAN*  *pds5::tetO2-DHFR-Myc1-PDS5::KAN*  *lacO repeat::URA*  *GFP-LacI::HIS3* |
| 2436 | *CEN IV::tetO(x448)::URA3 (2 kb from CEN IV) pURA3-tetR-GFP::LEU2 SPC42-Tomato::NAT Met-CDC20::URA*  *wpl1::HPH* |
| 2452 | *ubr1::GAL-HA-UBR1 (HIS3)*  *CMVp (tetR´-SSN6)::LEU2*  *trp1::tTA*  *SCC1-HA6::TRP1*  *pds5::tet02-DHFR-Myc18-PDS5::KAN* |
| 2455 | *ubr1::GAL-HA-UBR1 (HIS3)*  *CMVp (tetR´-SSN6)::LEU2*  *trp1::tTA*  *SCC1-HA6::TRP1*  *scc3::tet02-DHFR-Myc18-SCC3::KAN* |
| 2456 | *ubr1::GAL-HA-UBR1 (HIS3)*  *CMVp (tetR´-SSN6)::LEU2*  *trp1::tTA*  *SCC1-HA6::TRP1*  *scc3:tet02-DHFR-Myc18-SCC3::KAN*  *pds5::tet02-DHFR-Myc18-PDS5::KAN* |
| 2523 | *PDS5-HA6::HIS* |
| 2525 | *PDS5-HA6::HIS3 SCC1-Myc18::HIS3* |
| 2576 | *ubr1::GAL-HA-UBR1 (HIS3)*  *CMVp (tetR´-SSN6)::Leu2*  *trp1::tTA*  *PDS5-HA6::TRP* |
| 2577 | *ubr1::GAL-HA-UBR1 (HIS3)*  *CMVp (tetR´-SSN6)::LEU2*  *trp1::tTA*  *pds5::KAN::tetO2-DHFR-Myc18-PDS5-HA6::TRP* |
| 2578 | *ubr1::GAL-HA-UBR1 (HIS3)*  *CMVp (tetR´-SSN6)::Leu2*  *trp1::tTA*  *SCC3-HA6::TRP* |
| 2579 | *ubr1::GAL-HA-UBR1 (HIS3)*  *CMVp (tetR´-SSN6)::LEU2*  *trp1::tTA*  *scc3::KAN::tetO2-DHFR-Myc18-SCC3-HA6::TRP* |
| 2584 | *scc3::Myc9-SCC3* |
| 2587 | *pds5::Myc9-PDS5* |
| 2590 | *pds5::Myc9-PDS5-HA6:: TRP1* |
| 2601 | *pds5::Myc9-PDS5-HA6-ECO1(aa63-109)::NAT* |
| 2603 | *scc3::Myc9-SCC3-HA6::HIS* |
| 2608 | *scc3::Myc9-SCC3-HA6-ECO1(aa63-109)::NAT* |
| 10589 | *SCC1-Myc18::HIS3* |
| 12544 | *SCC3-HA6::HIS3 TetR-GFP-TAP::LEU2* |

*^a^*All strains are isogenic in the W303 background unless indicated otherwise and have the genotype *MATa* *ade2-1 trp1-1 can1-100 leu2-3,112, his3-11,15 ura3* GAL psi^+^

**Supplemental References**

S1 Fernius J, Hardwick KG (2007) Bub1 kinase targets Sgo1 to ensure efficient chromosome biorientation in budding yeast mitosis. *PLoS Genet* **3**(11)**:** e213

S2 Lu J, Kobayashi R, Brill SJ (1996) Characterization of a high mobility group 1/2 homolog in yeast. J Biol Chem 271(52): 33678-33685.

S3 Studier FW (2005) Protein production by auto-induction in high density shaking cultures. *Protein Expr Purif* **41**(1)**:** 207-234

S4 Yeh E, Haase J, Paliulis LV, Joglekar A, Bond L, Bouck D, Salmon ED, Bloom KS (2008) Pericentric chromatin is organized into an intramolecular loop in mitosis. *Curr Biol* **18**(2)**:** 81-90
