# Supplementary material for: Cohesin Rings Devoid of Scc3 and Pds5 Maintain Their Stable Association with the DNA
Source: PLoS Genet. 2012 Aug 9;8(8):e1002856. doi: 10.1371/journal.pgen.1002856 (PMC3415457; doi:10.1371/journal.pgen.1002856)
Supplement: Table S2 — Summary of ChIP-seq reads alignment against the Saccharomyces cerevisiae genome. (DOC) [file pgen.1002856.s015.doc]

Table S2. Summary of ChIP-seq reads alignment against the *Saccharomyces cerevisiae* genome.

|  | Number of reads from ChIP-Seq experiment | Reads mapped to Yeast genome | | Reads uniquely mapped to  Yeast genome | | Reads uniquely mapped to Yeast chromosomal genome | | | |
| --- | --- | --- | --- | --- | --- | --- | --- | --- | --- |
|  |  | Number | mapped  ratio  ( %) | Number | mapped  ratio  (%) | Number | mapped  ratio  ( %) | Average coverage | covered region (%) |
| Untagged | 33, 753, 633 | 13, 780, 131 | 40.83 | 13, 436, 401 | 39.8 | 13, 171, 595 | 39.02 | 46.21 | 95.62 |
| WT | 31, 232, 864 | 17, 312, 701 | 55.43 | 17, 156, 957 | 54.93 | 16, 806, 814 | 53.81 | 59.41 | 94.97 |
| *PDS5*-degron | 36, 054, 821 | 20, 785, 528 | 57.65 | 29, 428, 467 | 57.24 | 20, 198, 722 | 56.02 | 101.49 | 95.36 |
| *SCC3*-degron | 33, 880, 101 | 29, 531, 339 | 87.16 | 20, 096, 507 | 86.86 | 29, 039, 735 | 85.71 | 71.04 | 95.54 |
| *Δwpl1* | 32, 914, 388 | 20, 245, 837 | 61.51 | 20, 639, 515 | 61.06 | 20, 066, 865 | 60.97 | 69.14 | 95.59 |

Shown are the numbers of sequenced reads for five Chip-Seq libraries. Between 40-87% of the reads could be aligned to the *Saccharomyces cerevisiae* genome. To reduce the effect of contamination with human DNA, we also aligned the reads to the human genome and determined the reads uniquely mapping to the yeast genome. For analysis we only used the reads mapping to the chromosomal DNA (in particular, excluding the mitochondrial genome). The average reads coverage for the five libraries ranged from 46-101, covering 95-96% of the chromosomal part of the *Saccharomyces cerevisiae* genome.
